# Dust Rains Deliver Diverse Assemblages of Microorganisms to the Eastern Mediterranean

**DOI:** 10.1038/srep22657

**Published:** 2016-03-04

**Authors:** Ghida Nouhad Itani, Colin Andrew Smith

**Affiliations:** 1 Department of Biology, American University of Beirut, Beirut, Lebanon

## Abstract

Dust rains may be particularly effective at delivering microorganisms, yet their biodiversities have been seldom examined. During 2011 and 2012 in Beirut, Lebanon, 16 of 21 collected rainfalls appeared dusty. Trajectory modelling of air mass origins was consistent with North African sources and at least one Southwest Asian source. As much as ~4 g particulate matter, ~20 μg DNA, and 50 million colony forming units were found deposited per square meter during rainfalls each lasting less than one day. Sequencing of 93 bacteria and 25 fungi cultured from rain samples revealed diverse bacterial phyla, both Gram positive and negative, and Ascomycota fungi. Denaturing Gradient Gel Electrophoresis of amplified 16S rDNA of 13 rains revealed distinct and diverse assemblages of bacteria. Dust rain 16S libraries yielded 131 sequences matching, in decreasing order of abundance, Betaproteobacteria, Alphaproteobacteria, Firmicutes, Actinobacteria, Bacteroidetes, Cyanobacteria, Epsilonproteobacteria, Gammaproteobacteria, and Deltaproteobacteria. Clean rain 16S libraries yielded 33 sequences matching only Betaproteobacteria family Oxalobacteraceae. Microbial composition varied between dust rains, and more diverse and different microbes were found in dust rains than clean rains. These results show that dust rains deliver diverse communities of microorganisms that may be complex products of revived desert soil species and fertilized cloud species.

Deserts supply prodigious amounts of dust^1^ that affect many human concerns, including weather^2^, climate^3^, and health^4^, and dust is transported large distances^5^. Satellites allow comprehensive monitoring and supply dramatic imagery^6^, and with trajectory modelling^7^, help reveal origins and dispersal of desert dust. The largest sources of dust are in North Africa, and the Arabian Peninsula is a significant source^8^.

The mineral and microbial composition of desert dusts and their effects on downwind ecologies and human health has attracted much interest^9^,^10^,^11^,^12^,^13^. Aeolian dust supplies important mineral nutrients to aquatic ecosystems^3^,^14^. Particulate matter^4^ and pathogens^9^,^15^ are of greatest health concerns. In addition to dry deposition of desert dust, wet deposition occurs in the Mediterranean region and is known as dust rain, red rain, bloody rain, coloured rain, and muddy rain. Dust rains have been noted^16^ and natural origins posited^17^ since antiquity. In the modern era, the biogeochemical and ecological roles of dust rains attract scientific interest^18^,^19^,^20^.

Wet deposition of dust by rain is much less studied than dry deposition, and very few reports of microbial composition are available^21^,^22^. Aware of the widespread transport of microorganisms in aeolian dust^10^ and clouds^23^, having observed occasionally dramatic dust rains in Lebanon (see Supplementary Fig. S1), and considering that rain clouds could provide additional protection against desiccation and solar radiation and efficiently wash microorganisms into recipient soils, we wondered as to the diversity of microorganisms delivered to the eastern Mediterranean by dust rains.

Estimates suggest that each gram of arid soil contains as many as 10^9^ prokaryotes^24^ that may be aerosolized with soil particles. Estimates of bacteria in clouds^25^ exceed 10^5^ ml^−1^. Modern molecular biology offers tools to identify species in environmental samples by isolation of DNA, amplification of small subunit ribosomal DNA genes, and DNA sequencing^26^. Metagenomic approaches have revolutionized environmental microbiology^27^ and allow identification of species without culturing^28^,^29^, although biases may still exist^30^.

Here, we examined the microbial composition of dust rains falling in Beirut, Lebanon using total DNA extraction, solid media cultures, amplification of 16S and 18S rDNA, and sequencing.

## Results

### Amount of particulate matter, DNA, and CFU found in rains varied widely

During 2011 and 2012 in Beirut, Lebanon, 21 rainfalls were collected, of which 16 appeared to contain dust (appeared muddy) before filtration. Rainfalls were filtered and the residues analysed. The mass of particulate matter in each rain was measured and found to vary widely, ranging between 20 and 4,000 mg m^−2^ (Table 1). The characterisation of rains as dusty was subjective and did not relate directly to mass of particulate matter deposited in the collector, rather, the appearance of dustiness (tan or ochre and turbid) usually correlated to a high ratio of particulate matter to rain.

Total DNA was extracted from 19 rainfalls by bead-beating of residues. DNA masses ranged from 150 to ~20,000 ng m^−2^ (Table 1). The mass of particulate matter did not correlate well to that of DNA (n = 18, Spearman’s correlation = 0.33; p = 0.19). Colony forming units (CFU) were determined by plating dilutions on solid media and growth for 3 days at room temperature. Culturable microbes ranged widely from 200 thousand to 50 million CFU m^−2^ (Table 1). No strong relationships were observed between CFU and DNA mass (n = 18, Spearman’s correlation = 0.36; p = 0.14), and between CFU and mass of particulate matter (n = 18, Spearman’s correlation = 0.48; p = 0.04).

### Most dust rain backward trajectories passed over arid regions of North Africa

**Figure 1:**
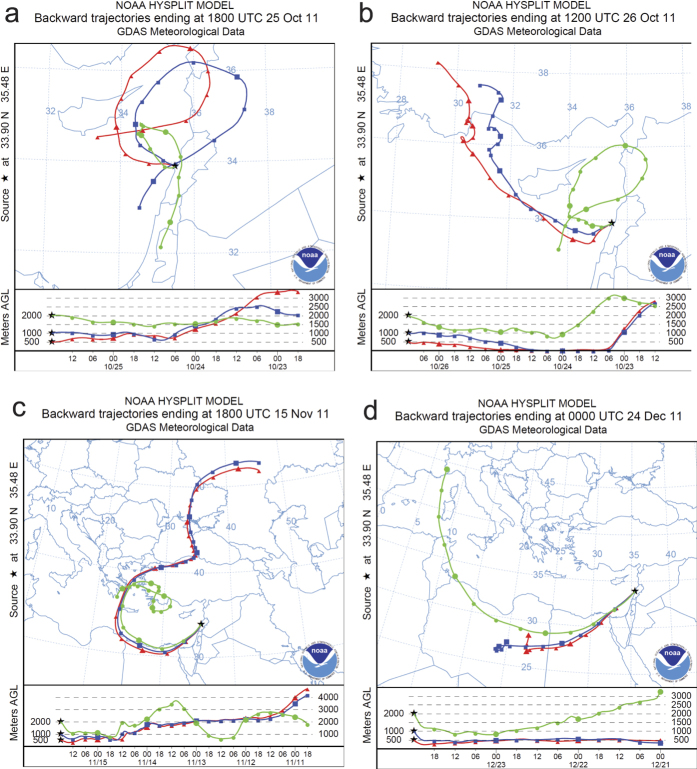
Wind Backward Trajectories of Four Selected Rains. (**a**) 25 October 2011. (**b**) 26 October 2011. (**c**) 15 November 2011. (**d**) 24 December 2011. Trajectories are for 72 hours ending at the described date and time. Red traces with triangles follow the air volume at 500 m; blue traces are for 1000 m; green traces are for 2000 m. Symbols mark 3-hour intervals with large symbols at 24-hour intervals. The images were obtained using the online HYSPLIT model from the US National Oceanic and Atmospheric Administration using Global Data Assimilation System meteorological data ( http://ready.arl.noaa.gov/HYSPLIT_traj.php). All trajectories are found in Supplementary Fig. S2.

The Hybrid Single Particle Lagrangian Integrated Trajectory (HYSPLIT) was used to trace air volumes backwards in time to predict plausible origins of solids and microorganisms in rains^7^. Most rains had trajectories consistent with acquisition of dust or soils from arid regions of North Africa, although one traced only from Southwest Asia, and several had mixed or uncertain trajectories (Fig. 1, see Supplementary Fig. S2).

### Dust rains contain diverse culturable microorganisms

Reasoner’s 2A agar was chosen for its ability to support growth of bacteria found in water and its relatively lower nutrient composition in the expectation that growth-rate differences between slow- and fast-growing microbes would be reduced. Rain cultures on solid media revealed live, diverse, and often pigmented colonies (see Supplementary Fig. S3). Cultures of two clean rains, 3 November 2011 and 15 November 2011, were notable for high CFU, less apparent diversity, and fewer pigmented colonies. Avoiding duplication of colonies of similar appearance, a few sample colonies were picked randomly from each rainfall for identification by amplification and sequencing of bacterial 16S rDNA and fungal 18S rDNA. Ninety-three sequences matched 16S database entries, comprising 5 phyla, including 3 classes of phylum Proteobacteria, in decreasing order of relative abundance: Actinobacteria (33/93), Firmicutes (19/93), Betaproteobacteria (16/93), Bacteroidetes (9/93), Gammaproteobacteria (9/93), Alphaproteobacteria (6/93), and Deinococcus-Thermus (1/93) (Table 2, see Supplementary Table S1, S2). No Bacteroidetes or Deinococcus-Thermus were cultured from clean rains. Twenty-five sequences matched 18S database entries, all from the phylum Ascomycota (Table 3). Ascomycota classes included Dothideo (17/25), Eurotiomycetes (6/25), and Sordariomycetes (2/25). Only Dothideomycetes were found in clean rain cultures, although only 3 cultures were sequenced (Table 3, see Supplementary Table S3).

### DGGE analysis of 16S rDNA shows diversity of bacteria in dust rains

**Figure 2:**
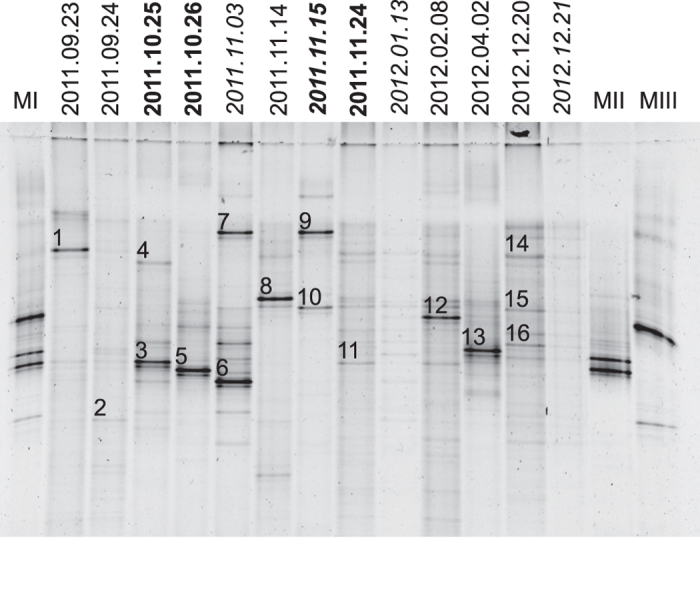
Denaturing Gradient Gel Electrophoresis of Bacterial 16S of Selected Rain Residues. Lanes are labelled by the date of the rain (YYYY.MM.DD). Clean rain headings are italicized, and headings of rains selected for greater sequencing are bold. Lane MI is a mix of *Escherichia coli, Bacillus cereus, Proteus vulgaris* and *Salmonella enterica*; lane MII is *E. coli* and *B. cereus*; Lane MIII is *P. vulgaris* and *S. enterica*. Prominent bands that were excised for sequencing are numbered directly above. The image was generated by Quantity One (version 4.6.3, Bio-Rad Laboratories) with adjustments in Photoshop CS6 (Adobe Systems).

In order to display the diversity of bacteria in dust rains including unculturable species, Denaturing Gradient Gel Electrophoresis (DGGE) was used to separate and visualize a 233 bp amplicon of the V3 variable region of 16S rDNA in 13 rainfalls (Fig. 2). Each rain presented a pattern of bands consistent with different and diverse assemblages of bacteria. Several samples were dominated by one or two prominent bands, and 16 bands were excised for sequencing, of which 9 yielded readable sequences. Bands 3, 6, and 7 yielded readable sequences closely matching Betaproteobacteria class Burkholderiales genera *Pelomonas*, *Ralstonia*, and *Herbaspirillum*, respectively (see Supplementary Table S4). Bands 5 and 9 were short, but matched Burkholderiales entries. Although the sequence of band 9 of clean rain of 15 November 2011 was short and of poorer quality, it aligned precisely with band 7 of clean rain of 3 November 2011 and matched *Herbaspirillum* as well as any other entry, suggesting it represents the same species as band 7 (Fig. 2). Other than the alignment of bands 7 and 9, no other shared bands were identified with confidence.

### Sequencing dust rain DNA reveals diverse compositions

Because the majority of bacteria would not have been cultured^28^,^29^, we made plasmid libraries from amplified 16S rDNA extracted from rain residues. Individual clones were sequenced from each rain to yield a total of 164 sequences (Table 2, see Supplementary Table S2). To understand how the microbiota of dust rains might be distinct from clean rains and dry depositions of desert dust, we selected 4 distinct rains for more extensive sequencing from their 16S libraries: one appearing to be clean rain and hail, 15 November 2011, one having a canonical North Africa backward trajectory, 24 December 2011, one of uncertain origin and high CFU, 25 October 2011, and one of uncertain origin and low CFU, 26 October 2011 (Fig. 1). Across 5 dates, 33 clean rain sequences displayed little diversity, matching only class Burkholderiales of Betaproteobacteria. In strong contrast, across 10 dates, dust rains displayed great diversity, with 101 of 131 having matches within 5 classes of Proteobacteria, and the remainder having matches to 7 Actinobateria, 6 Bacteriodetes, 4 Cyanobacteria, and 13 Firmicutes. In accord with the DGGE banding, each rain presented a different collection of bacterial sequences, and 3 sequenced DGGE bands from clean and dust rains matched the same genera as sequences found in their cloned 16S libraries (see Supplementary Table S4).

Fungal 18S rDNA was amplified from rain residues and yielded 35 sequences matching Ascomycota and Basidomycota (Table 3, see Supplementary Table S3). In clean rains were found only Basidomycota classes Agaricomycetes and Tremellomycetes and matches to uncultured eukaryotes. Interestingly, fungal sequences from the clean rain of 15 November 2011 matched only uncultured eukaryotes. Many dust rain 18S sequences matched Basidomycota classes Agaricomycetes and Tremellomycetes, and unlike clean rain sequences, many Ascomycota class Dothideomycetes. A few sequences matched other classes of Ascomycota and Basidomycota, and two matched uncultured eukaryotes.

**Figure 3:**
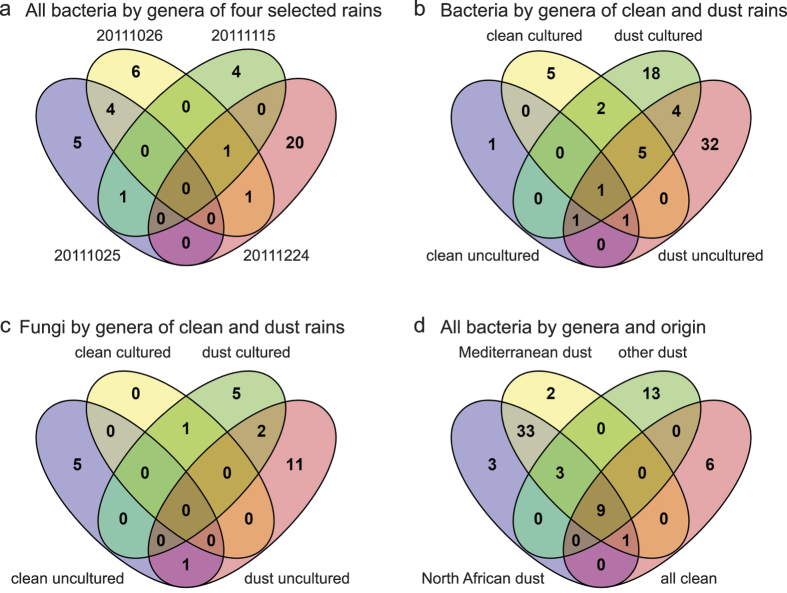
Comparisons of Rains. Venn diagrams showing number of specific and shared genera identified. (**a**) all identified bacterial genera of the four rains of more intense study (25 October 2011, 26 October 2011, 15 November 2011, and 24 December 2011). (**b**) Bacterial genera identified in combined clean rains and combined dust rains separated by culture-dependent and culture-independent isolation. (**c**) Fungal genera identified in combined clean rains and combined dust rains separated by culture-dependent and culture-independent isolation. (**d**) All identified bacteria categorized according to Table 1 as being dust rains with back-trajectories including North Africa, the Mediterranean, neither North Africa or the Mediterranean, or clean rains of all origins. Images were generated using Venny 2.1 ( http://bioinfogp.cnb.csic.es/tools/venny/index.html).

All sequences were categorized by genus, and rains were analyzed for specific and shared genera in different groupings (Fig. 3). Each rain selected for more 16S rDNA sequencing included genera specific to that rain (Fig. 3a). Interestingly, the rain of 24 December 2011, a dust rain with a backward trajectory passing over North Africa, had the greatest diversity, Chao1 index = 47.17 and Shannon index = 2.75, and the clean rain of 15 November 2011 had the least diversity, Chao1 index = 2 and Shannon index = 0.67 (Table 4). Analysis of separately combined clean and combined dust rain microbial composition with regard to sequences found in cultured or uncultured bacteria indicated both specific and shared genera. Dust rain contained more specific genera, and its diversity of uncultured genera was higher, Chao1 index = 88.1 and Shannon index = 3.38, than uncultured genera found in clean rain, Chao1 index = 4 and Shannon index = 1.07 (Table 4). Relatively few sequences were shared in cultured and uncultured groupings. Although many fewer fungal sequences were obtained, similar results were observed (Fig. 3c; Table 4). In order to determine whether microbial compositions correlated with backward trajectories, cultured and uncultured bacteria genera were categorized North African and dusty, Mediterranean and dusty, other origins and dusty, or clean of all origins (Fig. 3d). The North African and Mediterranean genera were probably shared, mostly because their backward-trajectories were shared, yet many genera found in dust rains of other and uncertain origins were specific.

## Discussion

Although the biogeochemistry of dust rains is an active area of research^18^,^19^, the microbiota of dust rains is much less studied. To our knowledge, this is the first study of the microbiota of dust rains in the eastern Mediterranean. Thus, our results may help address important gaps in the literature concerning airborne microbial communities, their origins, and possible ecological roles. Interestingly, we found each dust rain to display a different set of characteristics, that the composition of dust rain was significantly more diverse than that of clean rain, and that each rain appears to deliver a different assemblage of microorganisms. Dust rains with backward trajectories passing over North Africa or the Mediterranean shared relatively few bacterial genera with dust rains of other origins. The heaviest deposition within one day, of 4 g m^−2^, occurred in a light rain of 0.5 mm. This compares to yearly desert dust depositions estimated as 30-60 g m^−2^ yr^−1^ in the eastern Mediterranean^31^. Although the phenomenon of dust rain is often dramatic, heavy rainfalls depositing significant masses of particulate matter, especially at night, may be so diluted and washed that they pass without remark^20^. The distinction between clean and dust rains, stark as it sometimes appears, is too subjective to predict microbial composition and survival, and some clean rains also had significant particulate matter. Nonetheless, backward trajectories were consistent with most apparently dust rains acquiring their particulate matter from arid regions of North Africa and moisture from the Mediterranean Sea with subsequent orographic precipitation upon encountering Mount Lebanon^32^, similar to what has been observed in Anatolia^33^. Backward trajectories also suggest other mechanisms occur, including complex air mixtures and solids originating from arid regions in Southwest Asia, consistent with known weather patterns and dust sources in the region^11^,^31^,^33^,^34^. Importantly, clean rain backward trajectories avoided or skirted arid regions. Solid media cultures and DGGE analysis revealed most rains to have distinct microbial compositions, sometimes with prominent species. This is consistent with each rain acquiring microorganisms from different and possibly multiple sources integrated along complex paths that likely include non-arid terrestrial and marine surfaces^32^.

Most culture-based approaches likely miss a large proportion of species^28^,^29^, and our use of one type of solid media, R2A, limits ascertainable compositions. Thus, the CFU and diverse compositions observed in solid media cultures served primarily to indicate that many microorganisms had not been sterilized by desiccation or solar radiation (see Supplementary Fig. S3). Comparison of our observed CFU m^−2^ with published values of aerosolized desert dust^35^ CFU ranging as high as 15,000 m^−3^ and cloud water^25^ CFU approximately 400 ml^−1^ is difficult, but it suggests rains may scavenge large air volumes, or as discussed below, promote the growth of microorganisms resident in dust and clouds.

DGGE profiles, plate cultures, and sequence data all are consistent with each dust rain delivering a different composition of organisms. The selection imposed by culturing, especially on only one medium, may obscure distinctions between rains. Conversely, cultures revealed many Gram-positive bacteria that would otherwise not have been noted without extensive sequencing. Culture-independent analysis was attractive based on the significant masses of DNA extracted and difficulties of culturing most microorganisms^28^,^29^. Although it is difficult to rule out bias resulting from DNA extractions, amplification, and sequencing, most DNA samples were amplifiable and yielded readable sequences matching far more Alphaproteobacteria and Betaproteobacteria than did cultures. Interestingly, we were unable to amplify bacterial DNA from some light rains, consistent with DNA damage incurred from solar radiation or desiccation of dust before acquisition by clouds. Desert dust in the Mediterranean may sometimes be lofted and precipitated under continuous cloud cover^36^, and sometimes lofted before cloud formation^37^, the extremes of which may yield very different survival rates for many species.

Our observations are consistent with observed microbial compositions and variability of air^38^,^39^, rains and clouds^23^,^40^,^41^,^42^,^43^, desert dust^9^,^10^,^15^,^35^,^44^,^45^,^46^,^47^, and dust rains in the Alps^21^,^22^. Our data support the suggestion of Chuvochina *et al.*^21^ that delivered bacterial assemblages are affected more by the peculiarities of the transport and precipitation event more than by the source of particulate matter. Thus, each rain delivers a different, complex assemblage of microorganisms, and the microbial composition of dust rains needs further study. Our results encourage future studies to employ more sophisticated collection schemes, record meteorological data, conduct mineralogical analysis, use next-generation sequencing, and apply advanced statistical analysis.

In contrast to dust rains, the clean rain of 15 November 2011 appears to have a very low diversity (Table 4) and a high proportion of plant-associated members of family Oxalobacteraceae, consistent with its European backward trajectory and what is observed in clouds^23^,^40^,^41^,^43^. Interestingly, this torrential rain included hail and had the highest observed CFU, approximately 50,000 m^−2^ (~500 CFU ml^−1^). Hailstones have been observed to contain diverse bacteria, including Burkholderiales, which are often plant-associated and capable of nucleating ice, and can have CFU exceeding 5,000 ml^−1^ 43,48. The few library sequences of another clean rain on 3 November 2011 matched only members of order Burkholderiales, mostly *Ralstonia*, a common environmental genus^49^. Particularly interesting is the dominance of Oxalobacteraceae, a family that has been observed in the upper troposphere^41^. The sequences of cultured bacteria, DGGE bands, and 16S library sequences of clean rains on 3 November 2011 and 15 November 2011 all corroborate the dominant representation of *Ralstonia* and *Herbaspirillum* genera of order Burkholderiales in these rains.

In strong contrast to the clean rains, the dust rain of 24 December 2011, which had a canonical backward trajectory consistent with North African origins to its particulate matter, had sequences matching diverse bacterial phyla, including Gram-positive Actinobacteria and Firmicutes, as well as Bacteroidetes and several classes of Proteobacteria. Its diversity was high (Table 4), and it contained many specific bacterial genera (Fig. 3a). These observations, in particular the presence of Firmicutes, Actinobacteria, Firmicutues, Bacteroidetes, Alphaproteobacteria, and Burkholderiales (Betaproteobacteria), are similar to those observed in dust events^9^,^10^ and are consistent with soils from arid regions of North Africa^15^,^35^,^44^, including dust collected in the central and eastern Mediterranean^45^,^46^,^47^. Library and colony fungal sequences from this dust rain matched *Aspergillus*, *Cladosporidium*, and *Cryptococcus* fungal species, which have been found in dry dust depositions^9^,^35^,^45^. Intriguingly, many sequences match Gram-negative bacteria less typical of desert dust, such as aquatic Rhodobacteriaceae species, *Roseovarious crassostreae* and *Loktanella hongkongensis*, suggesting dust rains deliver species representing both desert dusts and rain.

In the uncertain-origin dust rains of 25 October 2011 and 26 October 2011, only sequences matching Alphaproteobacteria and Betaproteobacteria were found. Interestingly, they shared few bacterial genera with the dust rain of 24 December 2012. The lack of Gram-positive representation in 16S sequences in these two dust rains despite finding matches to Gram-positive bacteria in cultures emphasizes the utility of culturing when interests are directed to specific, culturable species. The representation of Burkholderiales and Sphingomonas sequences suggests a large proportion of non-dust species. Their different sequence compositions within Proteobacteria highlight the variability between events closely spaced in time.

Few reports of the microbial composition of dust rains are available^21^,^22^. In a study of three rains with and three without Saharan dust influence, Peter *et al.*^22^ find rains of Atlantic and continental origin dominated by Betaproteobacteria of genera *Massilia* and Sphingobacteria and rains with Saharan dust dominated by Gammaproteobacteria. Alphaproteobacteria and Gammaproteobacteria were found in both non-dust and dust influenced rains. *Massilia* dominated two of three dust influenced rains, and Bacilli were found only in dust rains. In contrast, we find few Gammaproteobacteria in dust rains, Massilia and Sphingobacteria only in dust rains, and clean rains dominated by Betaproteobacteria of Burkholderiaceae and Oxalobacteraceae. Our dust rains have similar Chao1 and Shannon values to the dust rains of Peter *et al.*^22^, yet our clean rains are much less diverse than our dust rains. A metagenomic study of Saharan dust in Alpine snow^21^, which included one dust rain in Grenoble, France, and a Saharan sand sample, also found great differences between samples. Actinobacteria (*Blastococcus*), Alphaproteobacteria (*Sphingomonas* and *Rubellimicrobium*), Bacteroidetes, Cyanobacteria, and Deinococcus-Thermus were particularly common. Compared to Chuvochina *et al.*^21^, we find more Betaproteobacteria and Firmicutes, yet fewer Deinococcus-Thermus. Our intensely studied dust rain of 24 December 2011 has Chao1 and Shannon values within the ranges they report for their libraries. Our results are not inconsistent with their findings, especially their observation that individual rains can deliver very different assemblages.

The data herein suggest that dust rains are an effective means by which microorganisms from desert dust and clouds are transported and delivered to surface habitats. More broadly, the culture and culture-independent sequencing, DGGE analysis, and solid-culture observations herein support the conclusion that each dust rain in the eastern Mediterranean likely delivers a unique collection of microorganisms, and that an understanding of their ecological significance, especially in expectation of natural and anthropogenic desertification and climate change, will require comprehensive examination. Extensive sampling of potential sources^50^,^51^, regular surveys, and standardization of methods will be needed.

One could imagine that transport in a raincloud would confer protection against desiccation and solar radiation and promote the survival of species lofted from arid soils. Less clear is whether dust rains offer a qualitatively different means of microbial transport beyond the efficiency of wet terrestrial deposition. Is dust rain microbiology a simple combination of that of rainclouds and desert dust? Fahlgren *et al.*^38^ speculate bacteria can grow in the atmosphere. Dust microorganisms can rapidly colonize aquatic habitats^52^ and dust provides nutrients to oligotrophic aquatic habitats^53^,^54^,^55^,^56^. Peter *et al.*^22^ find an approximately 100-fold increase in cell counts upon wet deposition of desert dust in Alpine lakes. Thus, dust rains may sustain and deliver microbiota that are complex products of revived desert species and fertilized cloud species. One might further speculate that the mixing of desert dust and clouds can create persistent habitats with water, soil, air, and light that propagate airborne microbial ecologies^12^.

## Methods

### Rain collection and processing

For each collection, a clean, new, polyethylene tarpaulin in a 2.1 square meter frame was placed on the southwest corner of the American University of Beirut biology building roof (33° 54′ 8′′ N, 35° 28′ 45′′ E, approximately 20 m above sea level) before expected rains. Each rain was collected 4-18 hour after falling and filtered through a 0.22 μm cellulose acetate membrane filter. No collection exceeded 24 h. One-sixteenth of each filter with residue was reserved for a glycerol stock, and the remainder divided into two portions, one portion stored in 1 ml of DNA extraction buffer EB (20 mM Tris·HCl pH 7.8, 50 mM ethylenediamine tetraacetic acid [EDTA], and 20 mM NaCl) in screw-cap, 2 ml, polypropylene microcentrifuge tubes for later extraction. The other portion was further divided in two, one portion separated from the filter, washed, and dried 24 h at 65 °C for mass measurement on an analytical balance, and the other stored in 0.5 ml EB.

### HYSPLIT backward trajectories

For each collected rainfall, wind backward trajectories were determined using the online Hybrid Single Particle Lagrangian Integrated Trajectory (HYSPLIT) model^7^ from the US National Oceanic and Atmospheric Administration using Global Data Assimilation System meteorological data ( http://ready.arl.noaa.gov/HYSPLIT_traj.php). Latitude (33.902° N) and longitude (35.479° E), total run time (at least 72 hrs), and altitudes (500 m, 1000 m, 2000 m) were entered for each rainfall date. The output was a map with the trajectory of the wind starting from the day of rainfall (Fig. 1, see Supplementary Fig. 2).

### Rain culturing

For each rainfall, the filter residue was stored in glycerol (20% final concentration) at −70 °C. Dilutions of each sample were plated on Reasoner´s 2A (R2A) agar using glass beads and cultured at room temperature. A small, non-random variety of colonies were restreaked for amplification and sequencing of bacterial 16S rDNA and fungal 18S rDNA as described below.

### DNA extraction and quantification

Total DNA was extracted from filters of rain residues using bead beating. To each 2 ml, screw-cap microcentrifuge tube containing filter and residue was added sodium dodecyl sulfate and RNaseA to final concentrations of 3% and 10 μl/ml, respectively. Spherical glass beads of 1 mm diameter were added to fill remaining volume. The samples were beaten at maximum speed on a Mini-Beadbeater-1 (Biospec Products, Bartlesville, OK, USA) for 2 min followed by placement on ice for 1 min. The beating was repeated twice, with an additional beating of 1 min. The tubes were centrifuged, supernatant collected, 500 μl of extraction buffer EB added, beaten for 2 min, and centrifuged. First and second supernatants of each sample were combined and extracted twice with phenol-chloroform-isoamyl alcohol (25:24:1) and once with chloroform. Glycogen (20 μg) was added to extracts, and they were precipitated with ethanol. Precipitated DNA samples were loaded alongside size and mass standards on 1% agarose (40 mM Tris, 20 mM acetic acid, and 1 mM EDTA, pH 8.0). The concentration of DNA was inferred by comparison to the intensity of the samples’ bands to standards.

### Denaturing gradient gel electrophoresis analysis

To assess the diversity of the microbial community in rain samples, a part of the V3 variable region in the 16S rRNA gene was amplified using universal denaturing gradient gel electrophoresis (DGGE) primers. P338FGC forward primer is 5′-cgcccgccgcgcgcggcgggcggggcgggggcacggggggCCTACGGGAGGCAGCAG-3′, and P518R reverse primer is 5′-ATTACCGCGGCTGCTGG-3′^57^. The lower case nucleotides represent the GC clamp, and the upper case nucleotides complement the target. Each 50 μl reaction of PCR contained 5 μl of DNA template 50 mM KCl, 10 mM Tris•HCl pH 8.3 @ 25 °C, 2.5 mM MgCl_2_, 0.2 mM deoxynucleoside triphosphates, 0.2 μM forward primer, 0.2 μM reverse primer, and 1.25 units of Taq polymerase. Amplification conditions used an initial denaturation at 95 °C for 2 min, followed by 35 cycles of denaturation at 95 °C for 30 sec, primer annealing at 55 °C for 30 sec, and extension at 72 °C for 90 sec. The final elongation step was extended to 20 min. Under these conditions, a single PCR product of 233 bp was obtained and subsequently gel purified. Authentic genomic DNA of *Escherichia coli*, *Bacillus cereus*, *Proteus vulgaris*, and *Salmonella enterica* were available in house and used to generate markers.

DGGE was performed using the DCode^TM^ Universal Mutation Detection System (Bio-Rad Laboratories, Hercules, CA, USA). A one mm thick gel containing 10% (w/v) polyacrylamide and a linear denaturing gradient of 30–70% was applied to separate 16S rRNA V3 PCR products (100% denaturant is defined as 7 M urea and 40% (v/v) formamide). The gels were prepared in running buffer (40 mM 10 mM Tris•HCl pH 8.0 @ 25 °C, 20 mM acetic acid, and 1 mM EDTA), which was also used as the electrophoresis buffer. Electrophoresis was applied for 16 hours at 60 °C, 75 V, and 50 mA. After electrophoresis, the gel was stained with Syber Green I (Molecular Probes, Eugene, OR, USA), and destained in running buffer. A tiff image was generated using a ChemiDoc XRS (Bio-Rad Laboratories) and Quantity One software (version 4.6.3, Bio-Rad Laboratories) from an 800 millisecond exposure with 254 nm transillumination and imported into Photoshop CS6 (Adobe Systems, San Jose, CA, USA), inverted, and the “adjust shadow input level” was increased from 0 to 230. Midtone and highlight input levels were left unadjusted at 1.00 and 255, respectively. Bright, easily visible bands from each lane on the DGGE gel were excised, and DNA was extracted by crushing and soaking. Extracts were amplified using the same PCR procedures as above using P338F (P338FGC without clamp: 5′-CCTACGGGAGGCAGCAG-3′) and P518R^57^.

### Amplification of small ribosomal subunit genes with bacterial and fungal primers

Bacterial 16S and fungal 18S rRNA genes were amplified from cultured colonies and total rain DNA using universal bacterial and fungal primers. FD1 forward bacterial primer is 5′-ccgaattcgtcgacaacAGAGTTTGATCCTGGCTCAG-3′, and RP2 reverse bacterial primer is 5′-cccgggatccaagcttACGGCTACCTTGTTACGACTT-3′^58^. EF3 forward fungal primer is 5′-ccgaattcgtcgaccTCCTCTAAATGACCAAGTTTG-3′, and EF4 reverse fungal primer is 5′-cccgggatccaagcttGGAAGGGGTGTATTTATTAG-3′^59^. The upper case nucleotides complement the target, and the lower case nucleotides represent linker sequences containing sites for the restriction enzymes EcoRI and HindIII for cloning. PCR conditions were as above, except primer annealing was at 52 °C or at 47 °C (for bacterial and fungal primers, respectively). The final elongation step was extended to 20 min. Under these conditions, a single PCR product of approximately 1.4 kb was obtained and subsequently gel purified.

### Molecular cloning

Products of total DNA amplified with FD1 and RP2 primers were digested with EcoRI and HindIII, ligated to a pcDNA3 plasmid with T4 DNA ligase, and transformed into chemically competent DH5α *Escherichia coli* using standard procedures^60^. Clones were screened by PCR for inserts, those without inserts discarded, and plasmid DNA was prepared.

### Sequencing and identification of DNAs

The sequencing primers were BGH-R (5′-TAGAAGGCACAGTCGAGG-3′) for plasmids (pcDNA3.1), 27F (5′-AGAGTTTGATCCTGGCTCAG-3′ [FD1 without restriction site extension]) for 16S PCR products, EF3 for 18S PCR products, and P338F and P518R for excised DGGE bands. Uncultured 16S sequence data have been submitted to GenBank under accession numbers KU740036 to KU740170.

Sequences obtained from plasmid sequencing were analysed by standard nucleotide Basic Local Alignment Search Tool (BLAST) at the National Center for Biotechnology Information (NCBI) database ( http://blast.ncbi.nlm.nih.gov/Blast.cgi) in the 16S ribosomal RNA sequences (Bacteria and Archaea) for bacterial amplicons and the non-redundant nucleotide collection for fungal amplicons. Matches with the highest percent sequence similarity to the query were recorded (see Supplementary Tables S2, S3 and S4).

### Venn diagrams and biodiversity indices

Sequences were categorized by aspects of interest and analyzed and displayed by use of Venny 2.1^61^ ( http://bioinfogp.cnb.csic.es/tools/venny/index.html). Chao1^62^ and Shannon indices were calculated using Microsoft Excel for sets of sequences originating from uncultured microbes only: sequences originating from cultures were not included because the avoidance of culturing colonies of similar appearance would impart bias to diversity calculations.

## Additional Information

**How to cite this article**: Itani, G. N. and Smith, C. A. Dust Rains Deliver Diverse Assemblages of Microorganisms to the Eastern Mediterranean. *Sci. Rep.* **6**, 22657; doi: 10.1038/srep22657 (2016).

## Supplementary Material

Supplementary Information

Supplementary Tables

## Figures and Tables

**Table 1:** Characterisation of rains.

Rain date^a^	Trajectory^b^	Observations^c^	Sources^d^	Exposure^e^, day	Rainfall, mm	PM^f^, mg/m^2^	DNA, ng/m^2^	CFU, 10^3^/m^2^
20110923	2011092310	muddy, dusty skies	SE, M, NAf	0.75	12	120	900	600
20110924	2011092400	muddy	SE, M	0.75	0.3	20	300	700
*20111001*	*2011093023*	*clean rain*	*WAs, M*	*0.75*	*20*	*100*	*NP*^e^	*7,000*
**20111025**	**2011102518**	**muddy, dusty skies**	**uncertain**	**0.65**	**20**	**300**	**5,000**	**3,000**
**20111026**	**2011102610**	**muddy**	**uncertain**	**0.15**	**5**	**30**	**3,000**	**300**
*20111103*	*2011110312*	*clean rain*	*SE, WAs, M*	*0.35*	*30*	*80*	*10,000*	*3,000*
20111114	2011111408	very muddy, dusty skies	uncertain	0.5	0.4	30	10,000	200
***20111115***	***2011111518***	***clean rain and hail***	***WAs, M, NAf***	***1***	***100***	***200***	***16,000***	***50,000***
**20111224**	**2011122400**	**muddy, dusty skies**	**SE, M, NAf**	**0.75**	**7**	**300**	**3000**	**4,000**
20120111	2012011112	muddy	E, M, NAf	1	17	80	NP^g^	6,000
*20120113*	*2012011300*	*clean rain*	*E, M, NAf*	*0.75*	*30*	*20*	*200*	*1,000*
20120208	2012020812	very muddy	E, M, NAf	1	6	500	3,000	4,000
20120215	2012021512	very muddy	NAf	1	5	300	11,000	700
20120314	2012031400	very muddy	SE, M, NAf	0.75	5	100	20,000	30,000
20120402	2012040200	very muddy	M, NAf, SWAs	0.75	1.0	300	9,000	500
20120420	2012042000	very muddy	E, NAf, M	0.75	0.3	130	300	1,000
20120430	2012043018	very muddy	SWAs	0.75	0.04	500	6,000	3,000
20120502	2012050215	very muddy	NAf, M, SWAs	0.75	0.5	4,000	12,000	1,600
20120529	2012052906	muddy, dusty skies	NAf, M	0.15	0.6	80	150	200
20121220	2012122000	muddy and hail	M, NAf	1	30	600	7,000	15,000
*20121221*	*2012122100*	*clean, smell of snow*	*SE, WAs, M*	*0.75*	*70*	*170*	*1,700*	*8,000*

^a^Rain date: the nominal date assigned to the rainfall, usually the calendar date of the morning after precipitation. Clean rain entries are italicized, and rains selected for more intense study are bold.

^b^Trajectory: the specific date and time used for the backward trajectory (format: YYYYMMDDHH).

^c^Observations: the general appearance of the rain water and weather when notable.

^d^Sources: wind backward trajectories passed over Europe (E), southern Europe (SE), the Mediterranean Sea (M), North Africa (NAf), western Asia (WAs), and southwestern Asia (SWAs). Most backward trajectories passed over multiple regions. Some did not yield coherent predictions and are labelled “uncertain.”

^e^No exposure time exceeded one day, and rain deposition was frequently of much shorter duration than exposure.

^f^PM: particulate matter.

^g^NP: Not processed; DNA was not extracted from this sample.

**Table 2:** Numbers of bacterial sequences obtained from cultures and uncultured total DNA.

Rain date	Matches to phyla in cultured bacteria^a^	Matches to phyla in uncultured bacteria^b^
**20110923**	3 Actino, 1 Beta, 1 Gamma	1 Alpha, 1 Cyano
**20110924**	2 Actino, 1 Deino	1 Alpha, 1 Beta, 1 Firmi
***20111001***	*3 Actino, 1 Firmi*	*NP*^c^
20111025	**ND**^d^	**5 Alpha, 23 Beta**
20111026	**1 Actino, 1 Firmi, 1 Gamma**	**11 Alpha, 28 Beta**
***20111103***	*2 Actino, 4 Beta, 1 Gamma*	*5 Beta*
**20111114**	2 Actino, 1 Bacter, 1 Beta, 1 Firmi	1 Actino, 3 Cyano
*20111115*	***1 Actino, 1 Alpha, 2 Beta, 1 Gamma***	***28 Beta***
20111224	**1 Actino, 1 Bacter, 2 Firmi**	**4 Actino, 13 Alpha, 2 Bacter, 2 Beta, 1 Delta, 3 Epsilon, 6 Firmi, 1 Gamma**
**20120111**	1 Actino, 1 Alpha, 1 Beta, 2 Firmi	NP
***20120113***	*2 Actino*	*0*^e^
**20120208**	1 Actino, 3 Bacter, 2 Firmi	1 Actino, 1 Alpha, 1 Bacter, 2 Beta, 3 Firmi
**20120215**	2 Bacter, 3 Beta,	3 Bacter
**20120314**	3 Actino, 1 Bacter, 1 Gamma,	2 Beta, 1 Gamma
**20120402**	1 Actino, 3 Beta, 2 Gamma,	1 Actino, 5 Beta, 3 Firmi
**20120420**	1 Actino, 1 Alpha, 1 Firmi, 1 Gamma	NA^f^
**20120430**	3 Actino, 1 Firmi	NA^f^
**20120502**	1 Actino, 1 Beta, 3 Firmi,	NA^f^
**20120529**	1 Actino, 1 Alpha, 1 Firmi	NA^f^
**20121220**	3 Actino, 1 Alpha, 1 Bacter, 1 Gamma	0^e^
***20121221***	*1 Actino, 1 Alpha, 4 Firmi*	0^e^

^a^Rain residues were cultured on Reasoner’s 2A agar and sample colonies identified by sequencing of amplified 16S rDNA. Identification abbreviations are Actino: Actinobacteria, Alpha: Alphaproteobacteria, Bacter: Bacterioidetes, Beta: Betaproteobacteria, Cyano: Cyanobacteria , Deino: Deinococcus-Thermus, Delta: Deltaproteobacteria, Epsilon: Epsilonproteobacteria, Firmi: Firmicutes, Gamma: Gammaproteobacteria.

^b^DNA was extracted from rain residues, 16S rDNA amplified, cloned, and sequenced. Identification abbreviations as in *a*.

^c^NP: Not processed.

^d^ND: Not determined.

^e^0: no readable sequences recovered.

^f^NA: Not amplifiable; PCR amplification was unsuccessful.

**Table 3:** Numbers of fungal sequences obtained from cultures and uncultured total DNA.

Rain date	Matches to classes in cultured fungi^a^	Matches to classes in uncultured fungi^b^
20110923	1 Dothideo, 1 Sordario	1 Dothideo
20110924	1 Dothideo	1 Agaricomycetes, 1 Basidiomycota
*20111001*	*1 Dothideo*	*NP*^c^
**20111025**	**ND**^d^	**1 Dothideo, 1 Basidiomycota, 1 uncultured**
**20111026**	**1 Dothideo**	**2 Basidiomycota**
*20111103*	*ND*^d^	*1 Agaricomycetes, 2 uncultured*
20111114	1 Dothideo	1 Dothideo
***20111115***	***ND***^d^	***4 uncultured***
**20111224**	**1 Dothideo, 1 Eurotio**	**2 Dothideo, 1 Tremellomycetes**
20120111	1 Eurotio, 1 Sordario	NP^e^
*20120113*	*1 Dothideo*	*2 Agaricomycetes, 1 Dothideo, 2 Tremellomycetes*
20120208	1 Dothideo	No clones found
20120215	ND^d^	NA^e^
20120314	ND^d^	NA^e^
20120402	1 Eurotio	2 Tremellomycetes
20120420	2 Dothideo	1 Agaricomycetes, 2 Dothideo, 1 Eurotio, 1 Malasseziomycetes, 2 Tremellomycetes, 1 uncultured
20120430	1 Dothideo	NA^e^
20120502	1 Dothideo, 1 Eurotio	NA^e^
20120529	2 Dothideo, 1 Eurotio	NA^e^
20121220	2 Dothideo, 1 Eurotio	0^f^
*20121221*	*1 Dothideo*	*2 Agaricomycetes*

^a^Rain residues were cultured on Reasoner’s 2A agar and sample colonies identified by sequencing of amplified 18S rDNA. Identification abbreviations are Dothidio: Dothideomycetes, Eurotio: Eurotiomycetes, Sordario: Sordariomycetes.

^b^DNA was extracted from rain residues, 18S rDNA amplified, cloned, and sequenced. Identification abbreviations as in *a*.

^c^NP: Not processed.

^d^ND: Not determined.

^e^NA: Not amplifiable. PCR amplification was unsuccessful.

^f^0: no clones found.

**Table 4:** Number of genera found in uncultured microbes and diversity indices.

Grouped sets of sequences^a^	Sequences^b^	Genera^c^	Chao1^d^	Shannon^e^
**20111025** uncultured bacteria	28	10	22.5	1.99
**20111026** uncultured bacteria	39	9	17	1.68
***20111115** uncultured bacteria*	*28*	*2*	*2*	*0.67*
**20111224** uncultured bacteria	32	19	47.17	2.75
*clean uncultured bacteria*	*33*	*4*	*4*	*1.07*
dust uncultured bacteria	131	44	88.1	3.38
*clean uncultured fungi*	*13*	*6*	*6.13*	*1.71*
dust uncultured fungi	22	14	39	2.46
dust NAf origins uncultured bacteria	57	27	60	3.10
dust Med orgins uncultured bacteria	57	30	75.13	3.15
dust other origins uncultured bacteria	71	18	30.25	2.47

^a^Sequences of uncultured bacteria and fungi were grouped by date of rain, rain classification as clean or dusty as in Table 1, and having back-trajectories passing over North Africa or the Mediterranean as in Table 1.

^b^The number of readable sequences in each category.

^c^The number of genera in each category.

^d^Chao1 index calculated with respect to genera.

^e^Shannon index calculated with respect to genera.
