# Supplementary material for: Dust Rains Deliver Diverse Assemblages of Microorganisms to the Eastern Mediterranean
Source: Sci Rep. 2016 Mar 4;6:22657. doi: 10.1038/srep22657 (PMC4778140; doi:10.1038/srep22657)
Supplement: Supplementary Information [file srep22657-s1.pdf]

Dust Rains Deliver Diverse Assemblages of Microorganisms to the Eastern Mediterranean

Ghida Nouhad Itani and Colin Andrew Smith*

Department of Biology, American University of Beirut, Beirut, Lebanon

*Corresponding author. *E-mail address*: colin.smith@aub.edu.lb; ++961-1-350 000 ext 3887; fax: ++961-1-350 000 ext 3888

a b

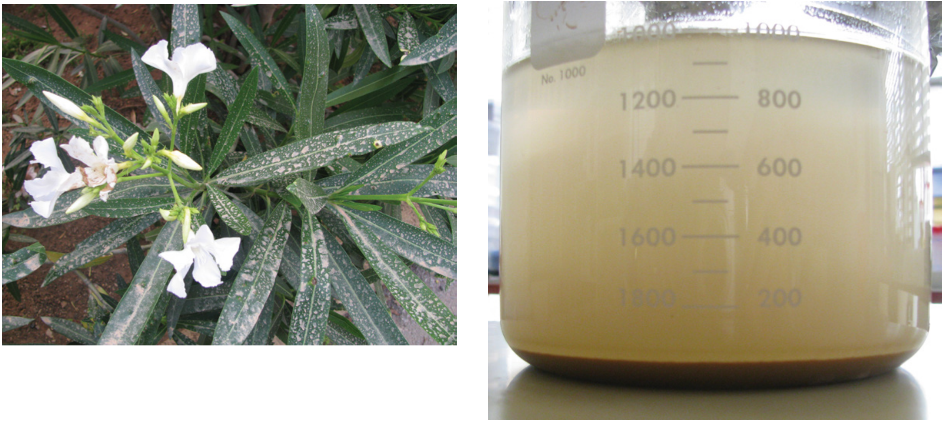

Dust Rains Deliver Diverse Assemblages of Microorganisms to the Eastern Mediterranean

Supplementary Figure S1. Dust rain in the Eastern Mediterranean. (a) Residue of a dust rain of May 2011 on *Nirsium oleander*. (b) A sample of May 2011 dust rain.

NOAA HYSPLIT MODEL
Backward trajectories ending at 1000 UTC 23 Sep 11
GDAS Meteorological Data

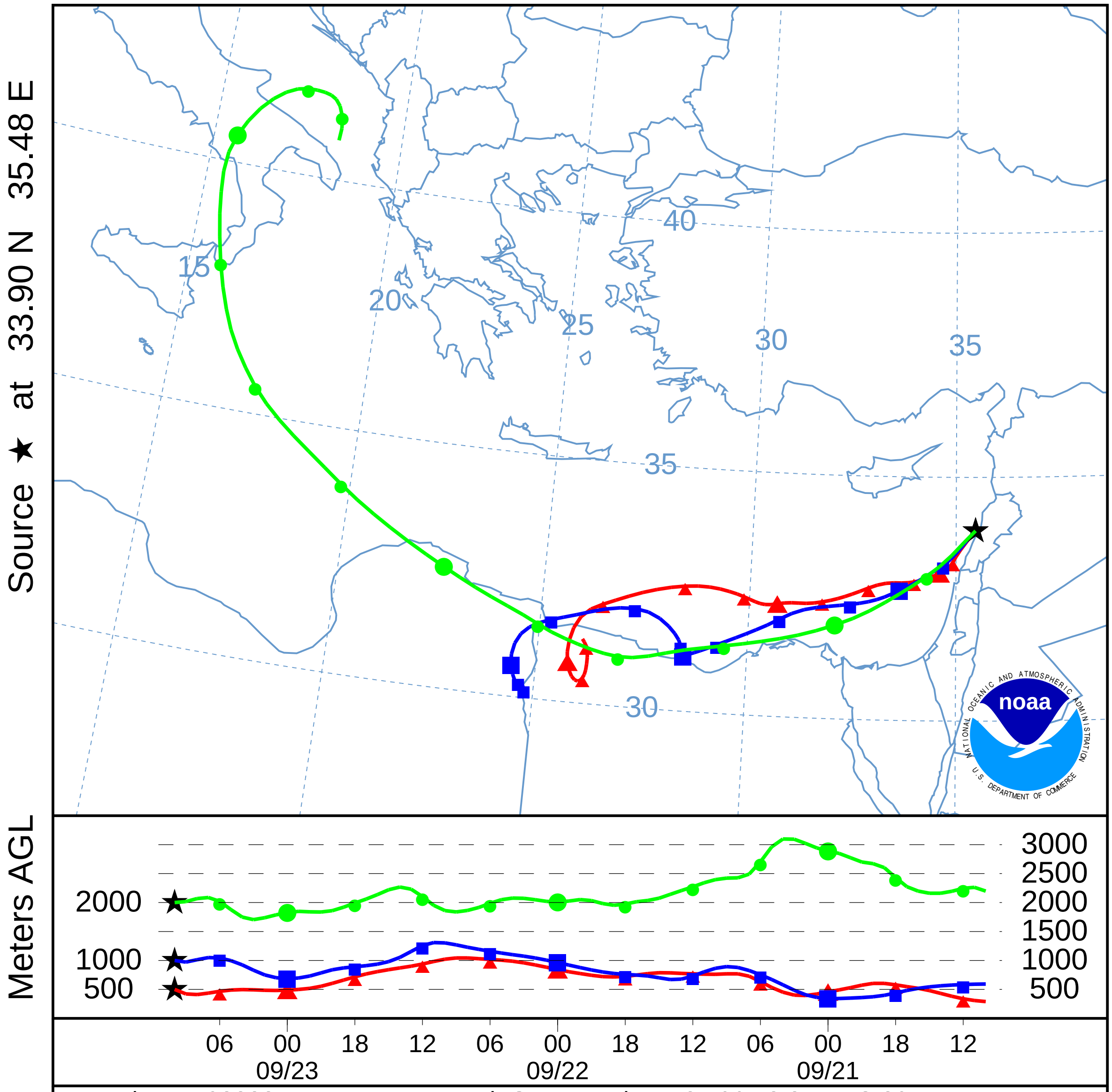

Job ID: 198833 Job Start: Wed Jun 24 06:53:27 UTC 2015
Source 1 lat.: 33.902000 lon.: 35.479000 hgts: 500, 1000, 2000 m AGL

Trajectory Direction: Backward Duration: 72 hrs
Vertical Motion Calculation Method: Model Vertical Velocity
Meteorology: 0000Z 22 Sep 2011 - GDAS1

NOAA HYSPLIT MODEL

Backward trajectories ending at 0000 UTC 24 Sep 11

GDAS Meteorological Data

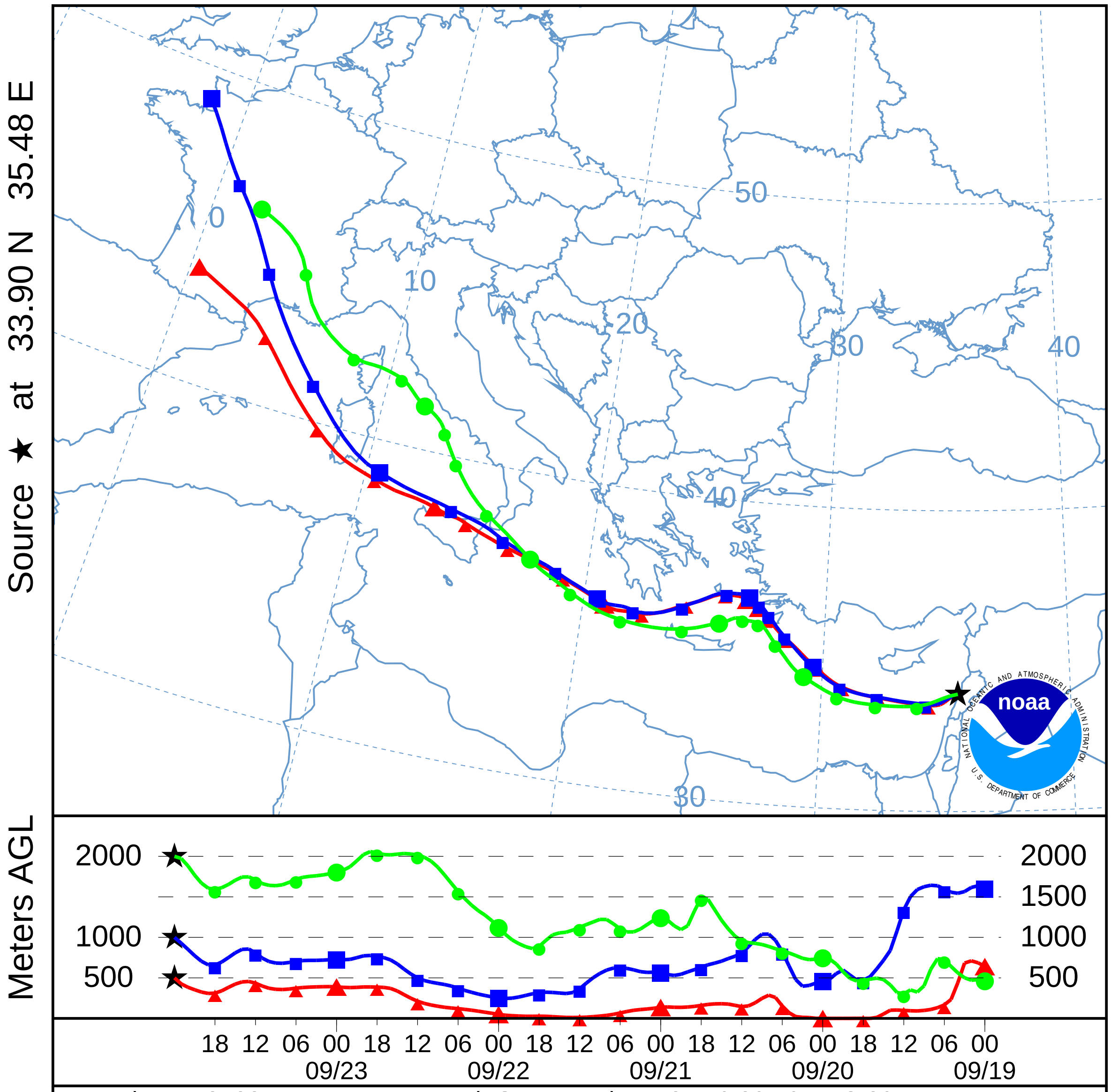

Job ID: 13522 Job Start: Wed Jun 24 10:20:59 UTC 2015
Source 1 lat.: 33.902000 lon.: 35.479000 hgts: 500, 1000, 2000 m AGL

Trajectory Direction: Backward Duration: 120 hrs
Vertical Motion Calculation Method: Model Vertical Velocity
Meteorology: 0000Z 22 Sep 2011 - GDAS1

NOAA HYSPLIT MODEL

Backward trajectories ending at 2300 UTC 30 Sep 11

GDAS Meteorological Data

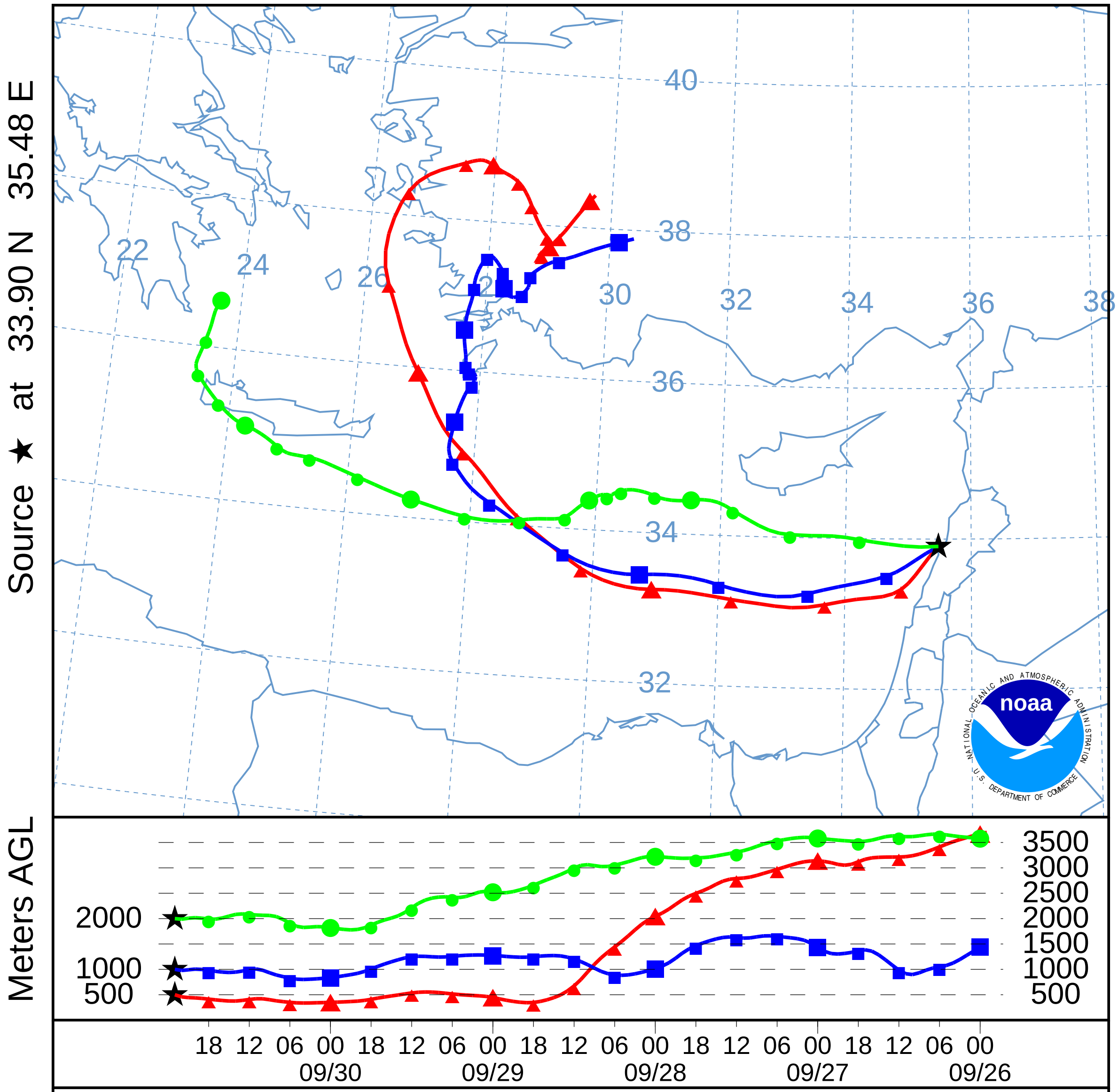

Job ID: 180912 Job Start: Tue Jun 23 10:54:43 UTC 2015

Source 1 lat.: 33.902000 lon.: 35.479000 hgts: 500, 1000, 2000 m AGL

Trajectory Direction: Backward Duration: 120 hrs

Vertical Motion Calculation Method: Model Vertical Velocity

Meteorology: 0000Z 29 Sep 2011 - GDAS1

NOAA HYSPLIT MODEL

Backward trajectories ending at 1800 UTC 25 Oct 11

GDAS Meteorological Data

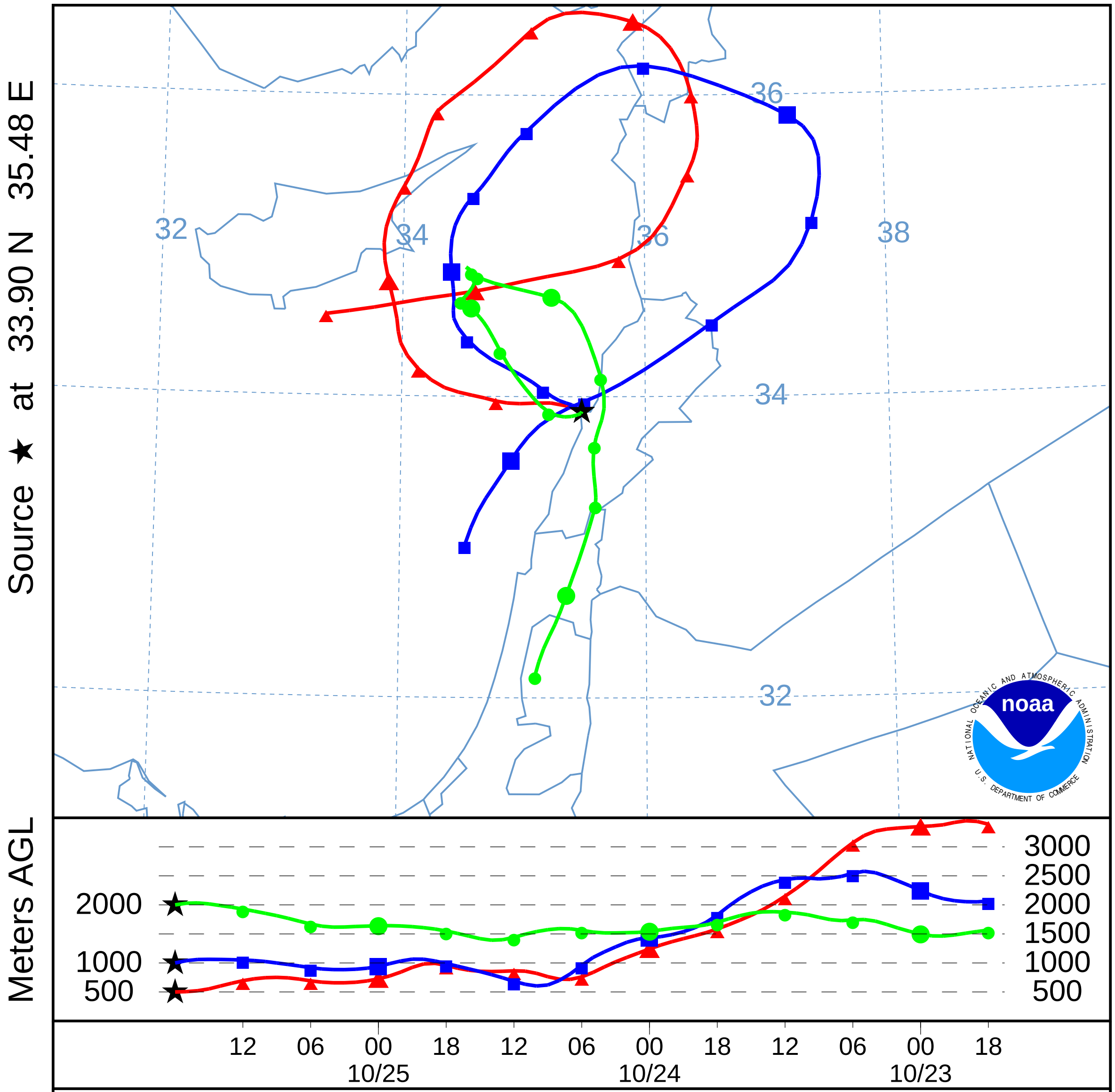

Job ID: 180316 Job Start: Tue Jun 23 10:10:13 UTC 2015
Source 1 lat.: 33.902000 lon.: 35.479000 hgts: 500, 1000, 2000 m AGL

Trajectory Direction: Backward Duration: 72 hrs
Vertical Motion Calculation Method: Model Vertical Velocity
Meteorology: 0000Z 22 Oct 2011 - GDAS1

NOAA HYSPLIT MODEL

Backward trajectories ending at 1200 UTC 26 Oct 11

GDAS Meteorological Data

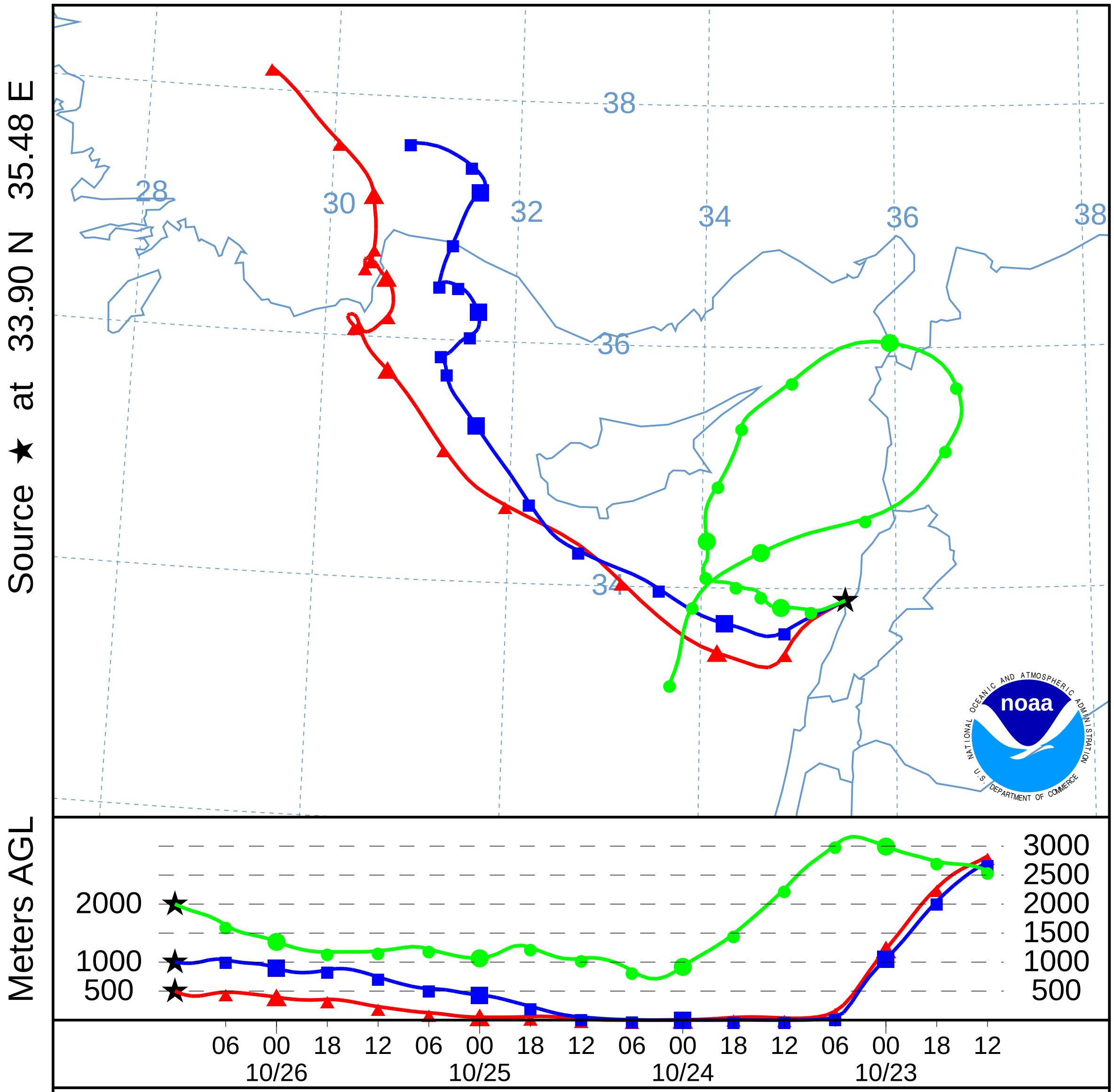

Job ID: 180657 Job Start: Tue Jun 23 10:37:31 UTC 2015
Source 1 lat.: 33.902000 lon.: 35.479000 hgts: 500, 1000, 2000 m AGL

Trajectory Direction: Backward Duration: 96 hrs
Vertical Motion Calculation Method: Model Vertical Velocity
Meteorology: 0000Z 22 Oct 2011 - GDAS1

NOAA HYSPLIT MODEL

Backward trajectories ending at 1200 UTC 03 Nov 11

GDAS Meteorological Data

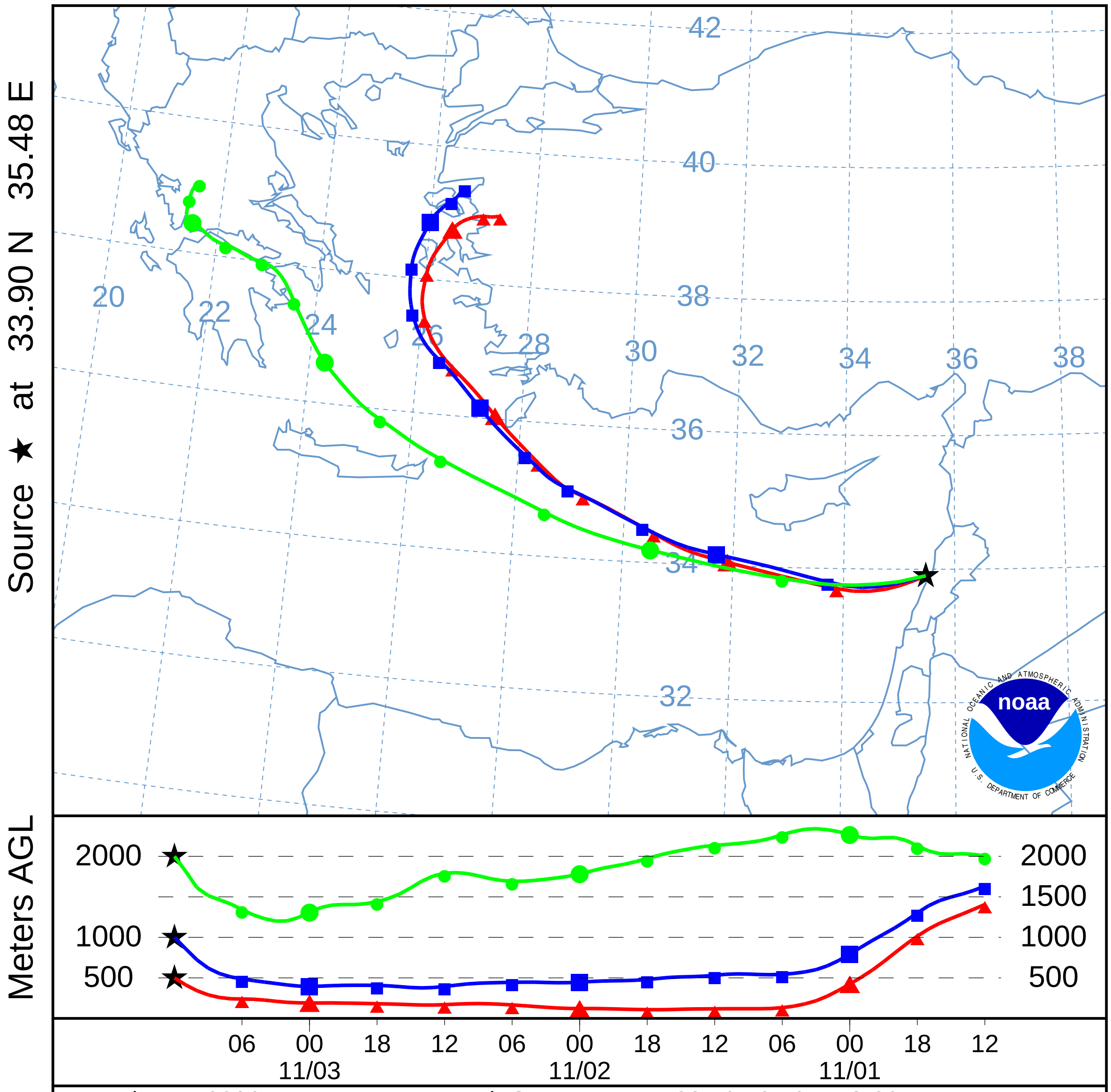

Job ID: 180865 Job Start: Tue Jun 23 10:50:49 UTC 2015
Source 1 lat.: 33.902000 lon.: 35.479000 hgts: 500, 1000, 2000 m AGL

Trajectory Direction: Backward Duration: 72 hrs
Vertical Motion Calculation Method: Model Vertical Velocity
Meteorology: 0000Z 1 Nov 2011 - GDAS1

NOAA HYSPLIT MODEL
Backward trajectories ending at 0800 UTC 14 Nov 11
GDAS Meteorological Data

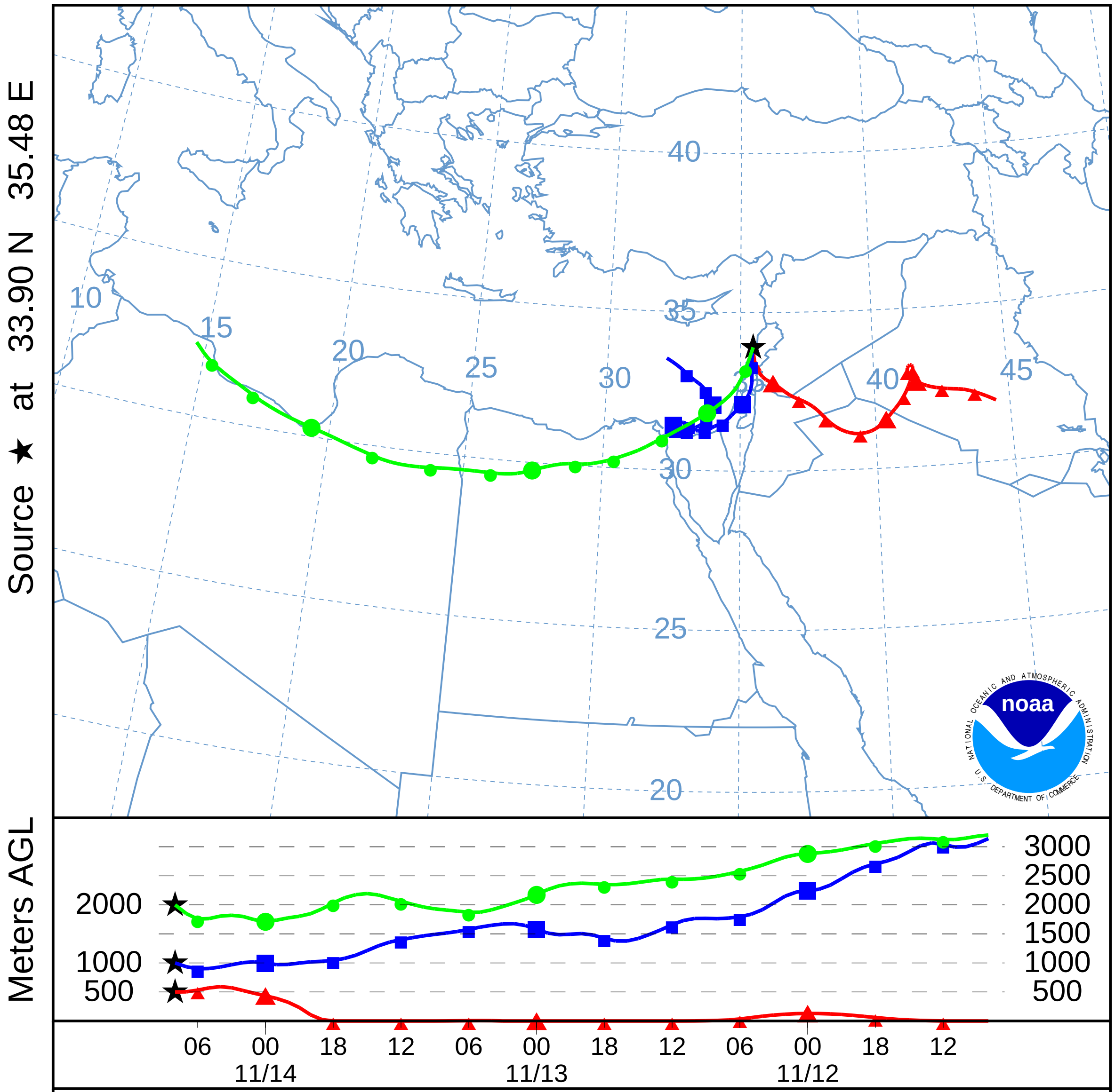

Job ID: 180953 Job Start: Tue Jun 23 10:57:38 UTC 2015
Source 1 lat.: 33.902000 lon.: 35.479000 hgts: 500, 1000, 2000 m AGL

Trajectory Direction: Backward Duration: 72 hrs
Vertical Motion Calculation Method: Model Vertical Velocity
Meteorology: 0000Z 8 Nov 2011 - GDAS1

NOAA HYSPLIT MODEL
Backward trajectories ending at 1800 UTC 15 Nov 11
GDAS Meteorological Data

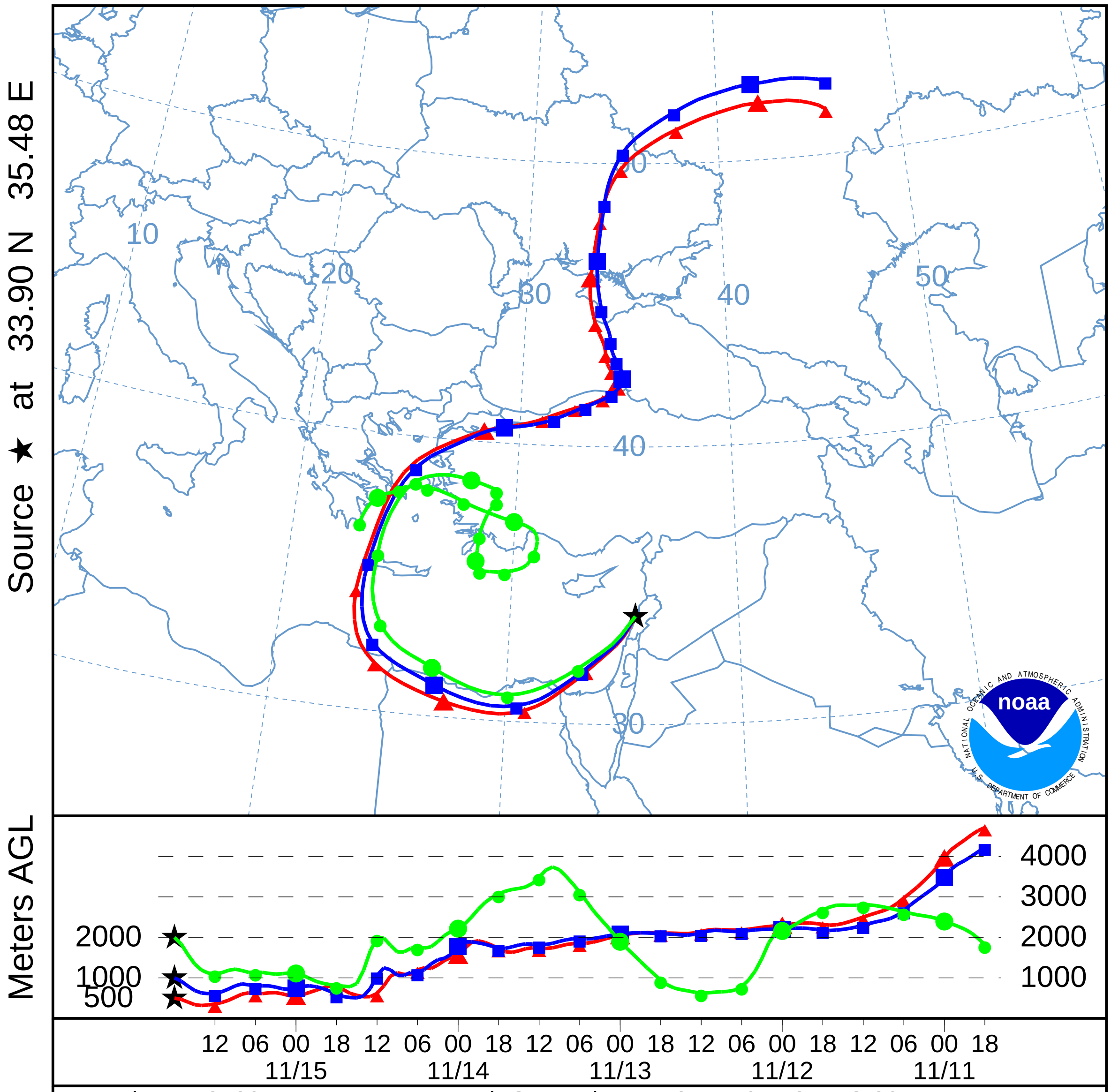

Job ID: 125897 Job Start: Thu Jun 25 11:01:43 UTC 2015
Source 1 lat.: 33.902000 lon.: 35.479000 hgts: 500, 1000, 2000 m AGL

Trajectory Direction: Backward Duration: 120 hrs
Vertical Motion Calculation Method: Model Vertical Velocity
Meteorology: 0000Z 15 Nov 2011 - GDAS1

NOAA HYSPLIT MODEL

Backward trajectories ending at 0000 UTC 24 Dec 11

GDAS Meteorological Data

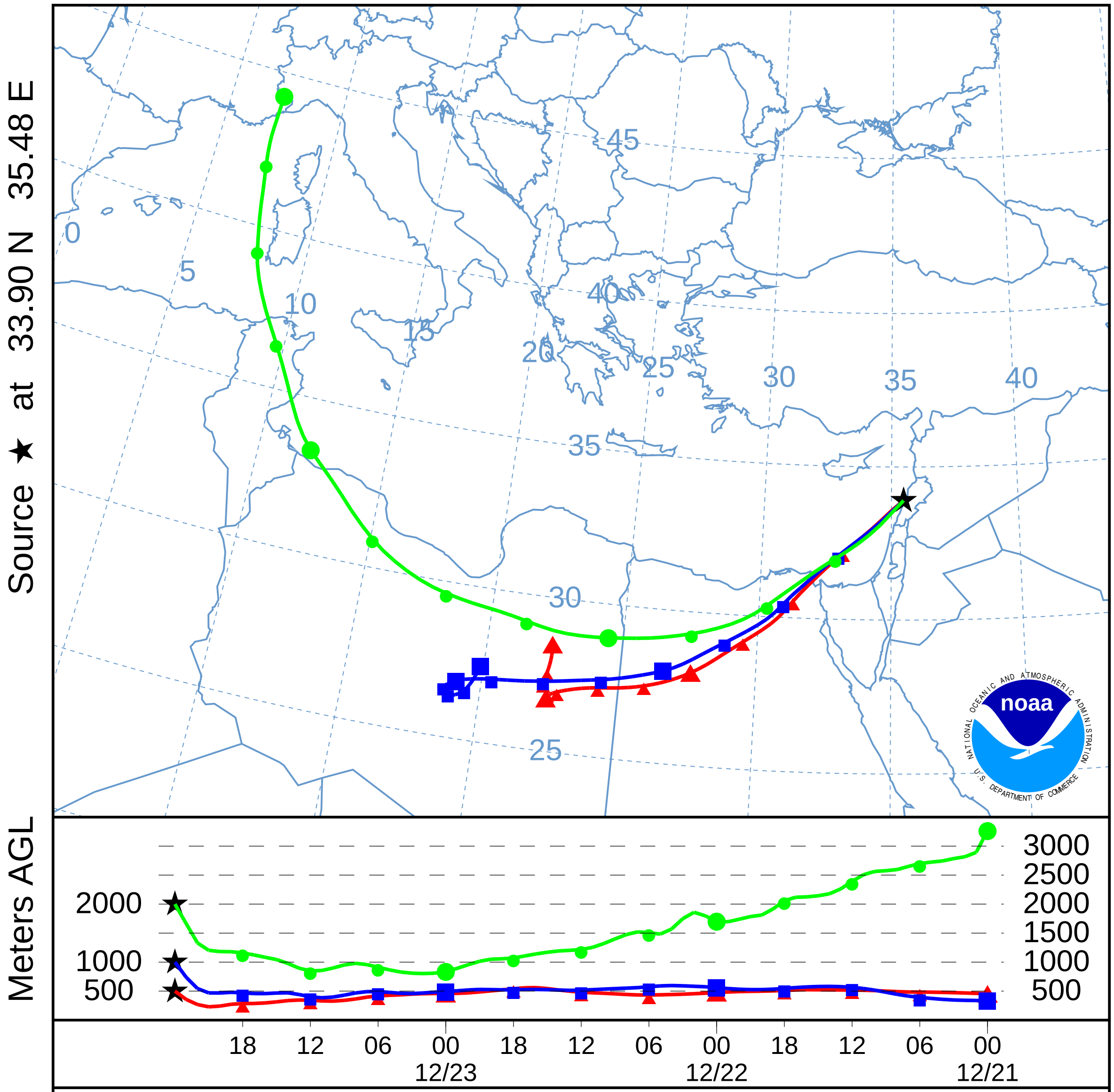

Job ID: 181137 Job Start: Tue Jun 23 11:14:04 UTC 2015
Source 1 lat.: 33.902000 lon.: 35.479000 hgts: 500, 1000, 2000 m AGL

Trajectory Direction: Backward Duration: 72 hrs
Vertical Motion Calculation Method: Model Vertical Velocity
Meteorology: 0000Z 22 Dec 2011 - GDAS1

NOAA HYSPLIT MODEL

Backward trajectories ending at 1200 UTC 11 Jan 12

GDAS Meteorological Data

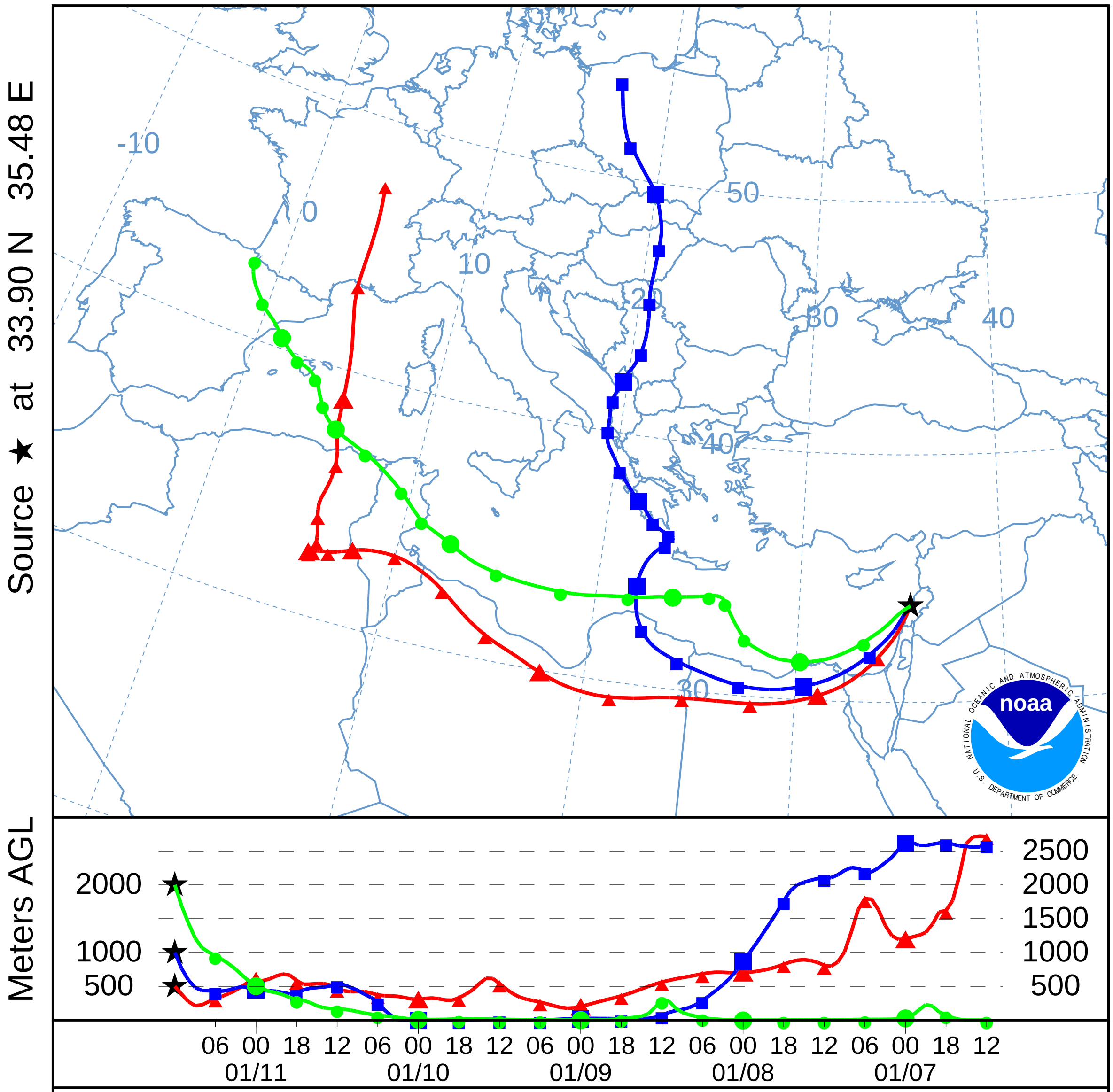

Job ID: 181186 Job Start: Tue Jun 23 11:19:07 UTC 2015
Source 1 lat.: 33.902000 lon.: 35.479000 hgts: 500, 1000, 2000 m AGL

Trajectory Direction: Backward Duration: 120 hrs
Vertical Motion Calculation Method: Model Vertical Velocity
Meteorology: 0000Z 8 Jan 2012 - GDAS1

NOAA HYSPLIT MODEL

Backward trajectories ending at 0000 UTC 13 Jan 12

GDAS Meteorological Data

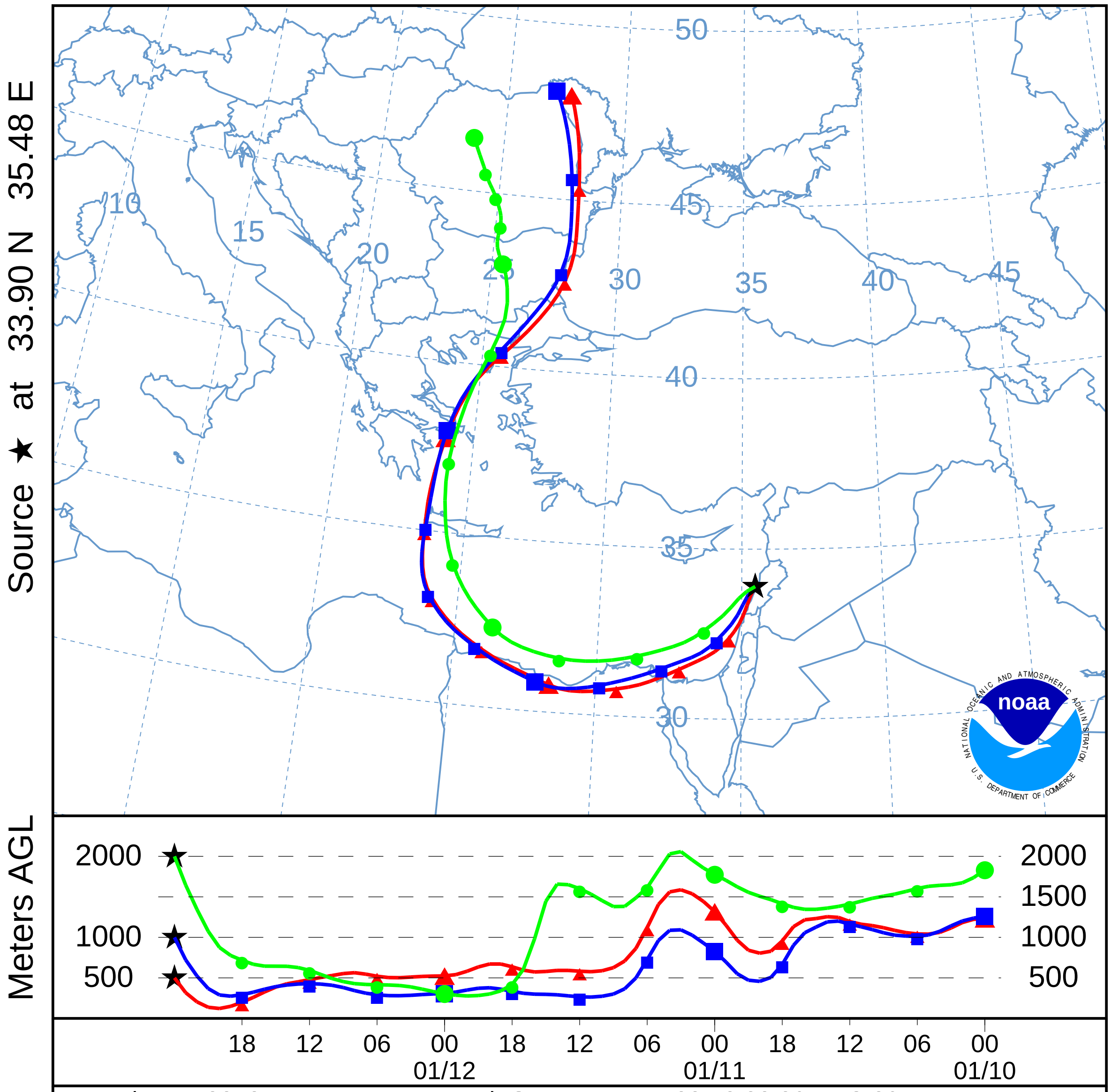

Job ID: 182781 Job Start: Tue Jun 23 12:33:30 UTC 2015
Source 1 lat.: 33.902000 lon.: 35.479000 hgts: 500, 1000, 2000 m AGL

Trajectory Direction: Backward Duration: 72 hrs
Vertical Motion Calculation Method: Model Vertical Velocity
Meteorology: 0000Z 8 Jan 2012 - GDAS1

NOAA HYSPLIT MODEL
Backward trajectories ending at 1200 UTC 08 Feb 12
GDAS Meteorological Data

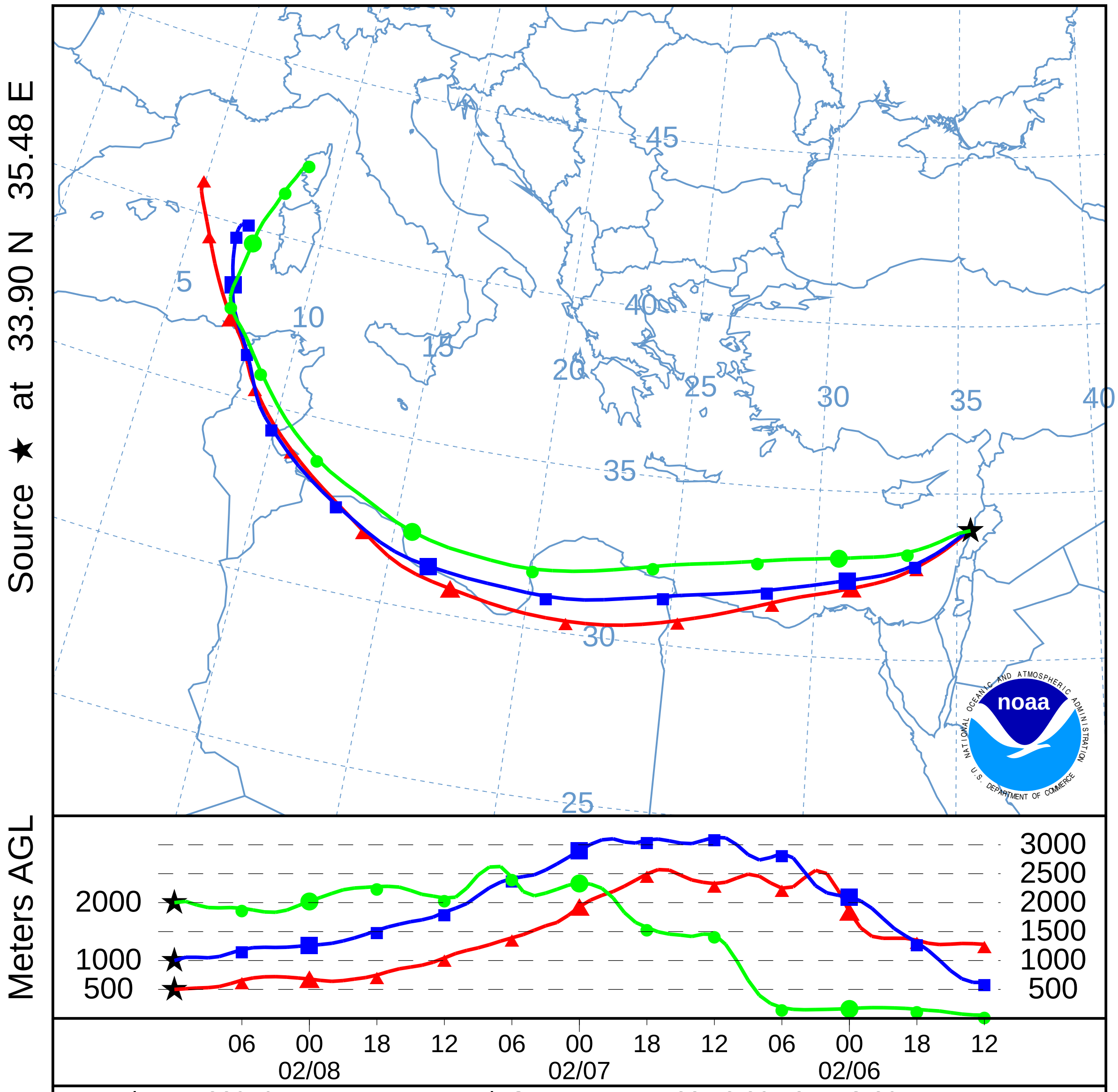

Job ID: 182940 Job Start: Tue Jun 23 12:39:52 UTC 2015
Source 1 lat.: 33.902000 lon.: 35.479000 hgts: 500, 1000, 2000 m AGL

Trajectory Direction: Backward Duration: 72 hrs
Vertical Motion Calculation Method: Model Vertical Velocity
Meteorology: 0000Z 8 Feb 2012 - GDAS1

NOAA HYSPLIT MODEL

Backward trajectories ending at 1200 UTC 15 Feb 12

GDAS Meteorological Data

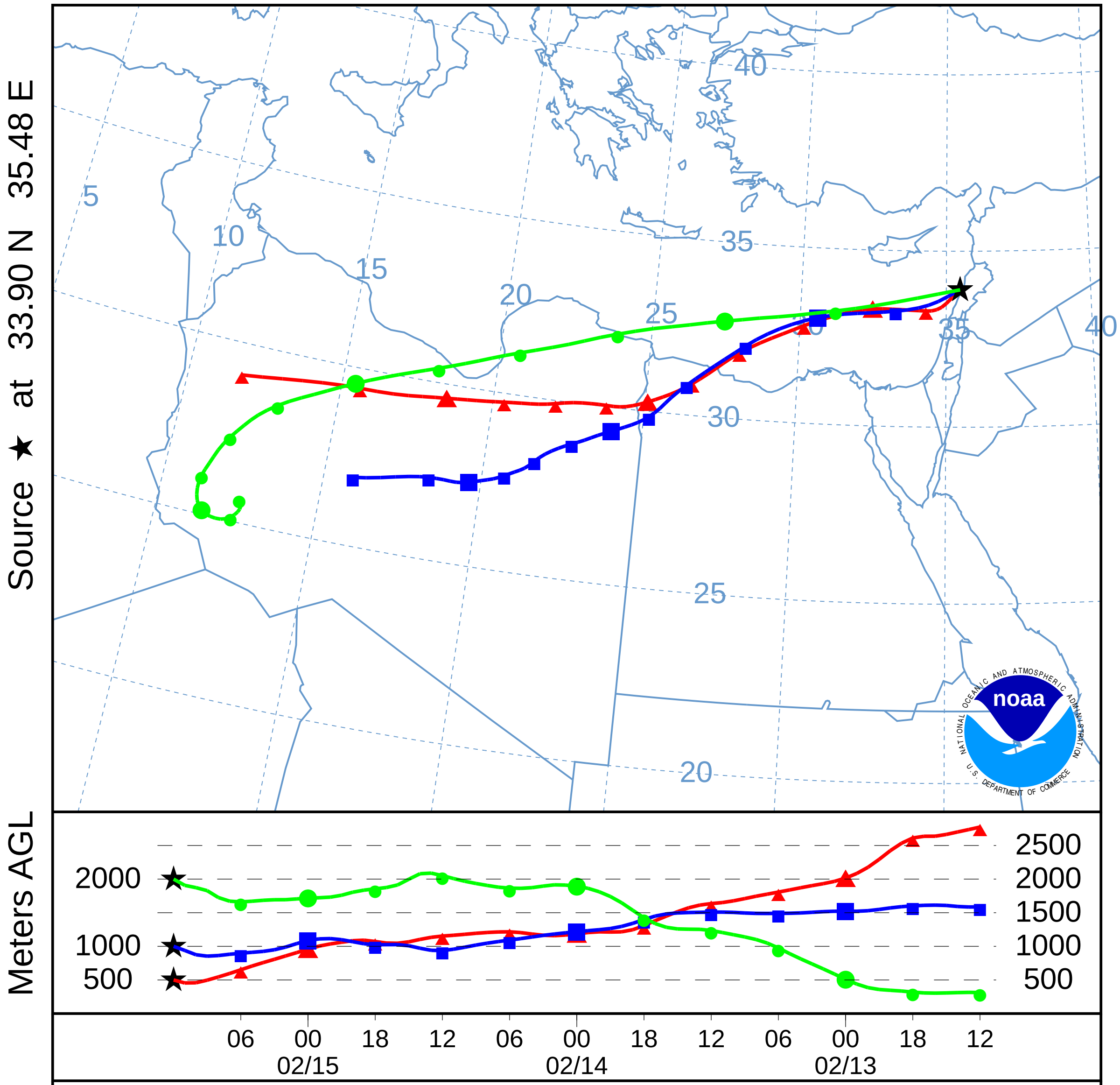

Job ID: 183023 Job Start: Tue Jun 23 12:44:04 UTC 2015
Source 1 lat.: 33.902000 lon.: 35.479000 hgts: 500, 1000, 2000 m AGL

Trajectory Direction: Backward Duration: 72 hrs
Vertical Motion Calculation Method: Model Vertical Velocity
Meteorology: 0000Z 15 Feb 2012 - GDAS1

NOAA HYSPLIT MODEL

Backward trajectories ending at 0000 UTC 14 Mar 12

GDAS Meteorological Data

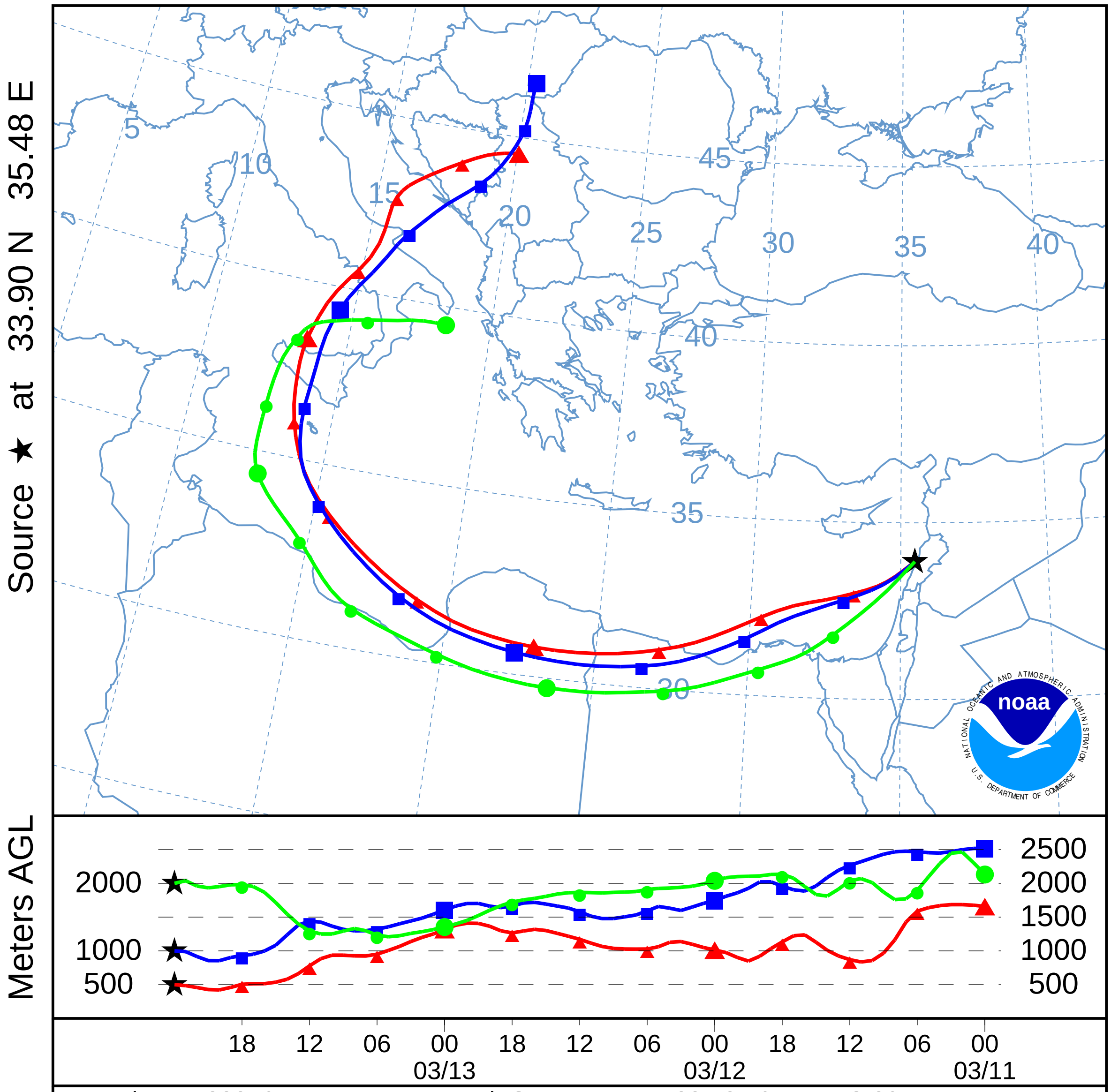

Job ID: 183246 Job Start: Tue Jun 23 12:56:47 UTC 2015
Source 1 lat.: 33.902000 lon.: 35.479000 hgts: 500, 1000, 2000 m AGL

Trajectory Direction: Backward Duration: 72 hrs
Vertical Motion Calculation Method: Model Vertical Velocity
Meteorology: 0000Z 8 Mar 2012 - GDAS1

NOAA HYSPLIT MODEL
Backward trajectories ending at 0000 UTC 02 Apr 12
GDAS Meteorological Data

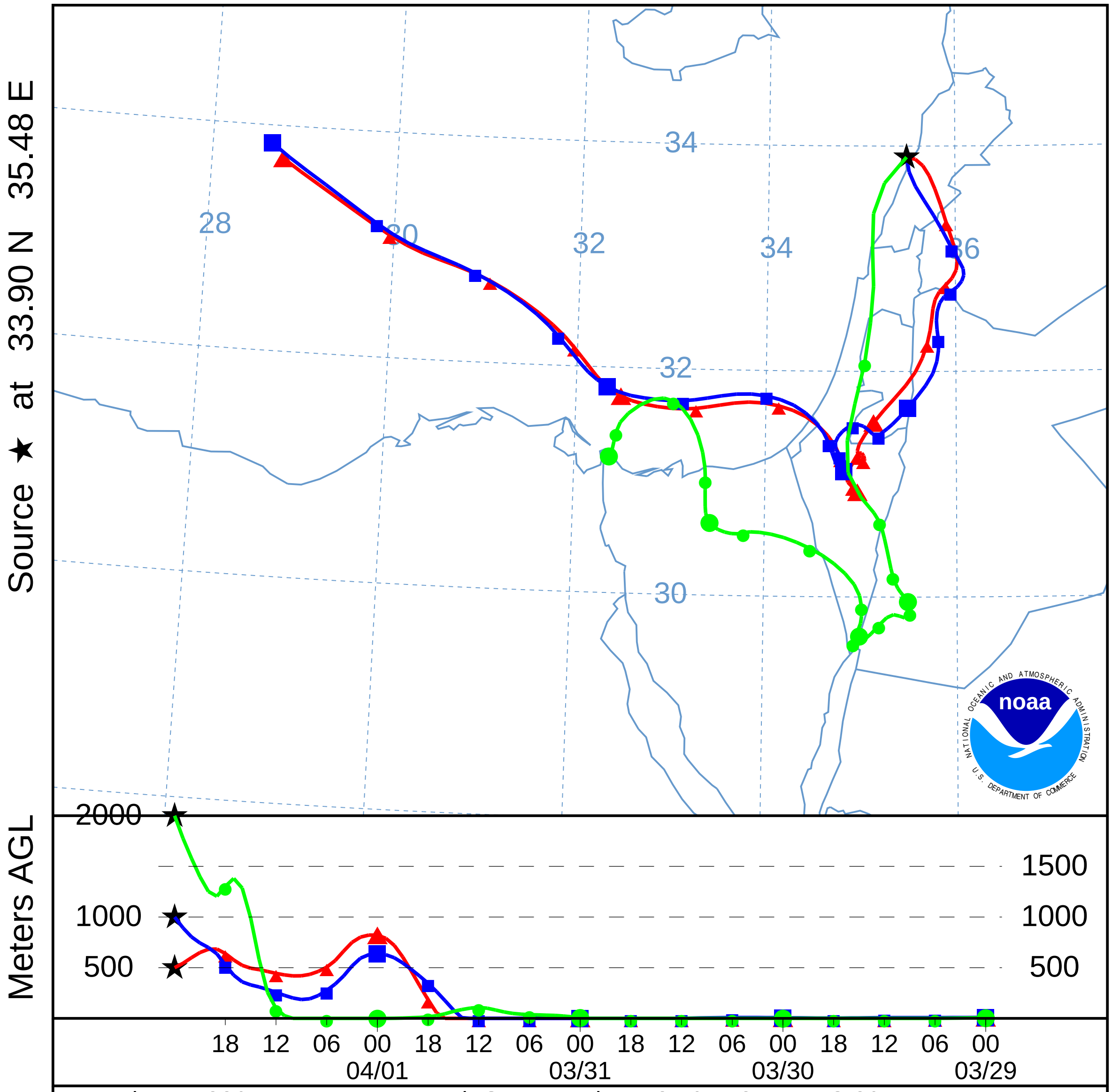

Job ID: 1826 Job Start: Wed Jun 24 07:52:47 UTC 2015
Source 1 lat.: 33.902000 lon.: 35.479000 hgts: 500, 1000, 2000 m AGL

Trajectory Direction: Backward Duration: 96 hrs
Vertical Motion Calculation Method: Model Vertical Velocity
Meteorology: 0000Z 1 Apr 2012 - GDAS1

NOAA HYSPLIT MODEL
Backward trajectories ending at 0000 UTC 20 Apr 12
GDAS Meteorological Data

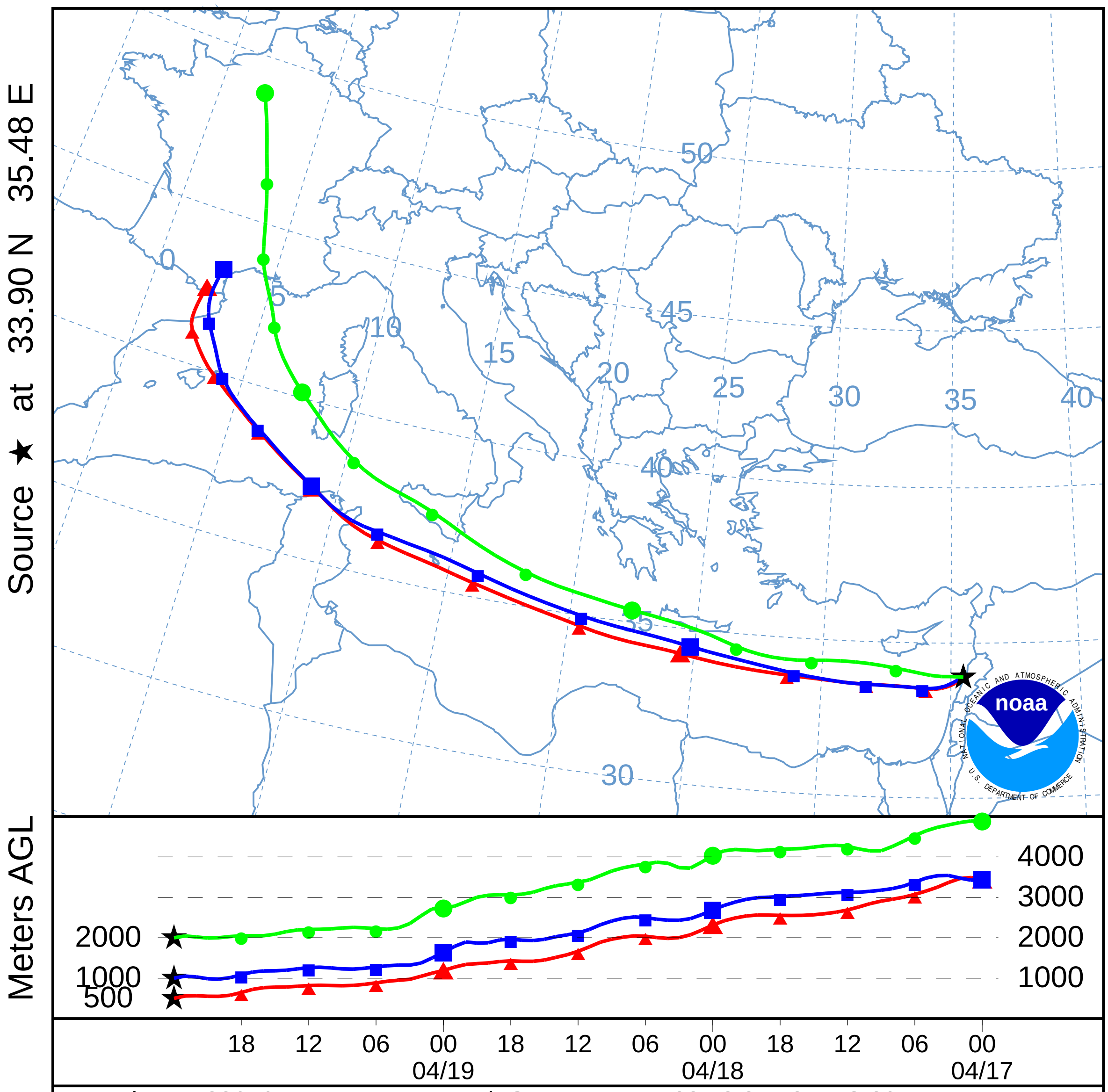

Job ID: 183676 Job Start: Tue Jun 23 13:21:53 UTC 2015
Source 1 lat.: 33.902000 lon.: 35.479000 hgts: 500, 1000, 2000 m AGL

Trajectory Direction: Backward Duration: 72 hrs
Vertical Motion Calculation Method: Model Vertical Velocity
Meteorology: 0000Z 15 Apr 2012 - GDAS1

NOAA HYSPLIT MODEL
Backward trajectories ending at 1800 UTC 30 Apr 12
GDAS Meteorological Data

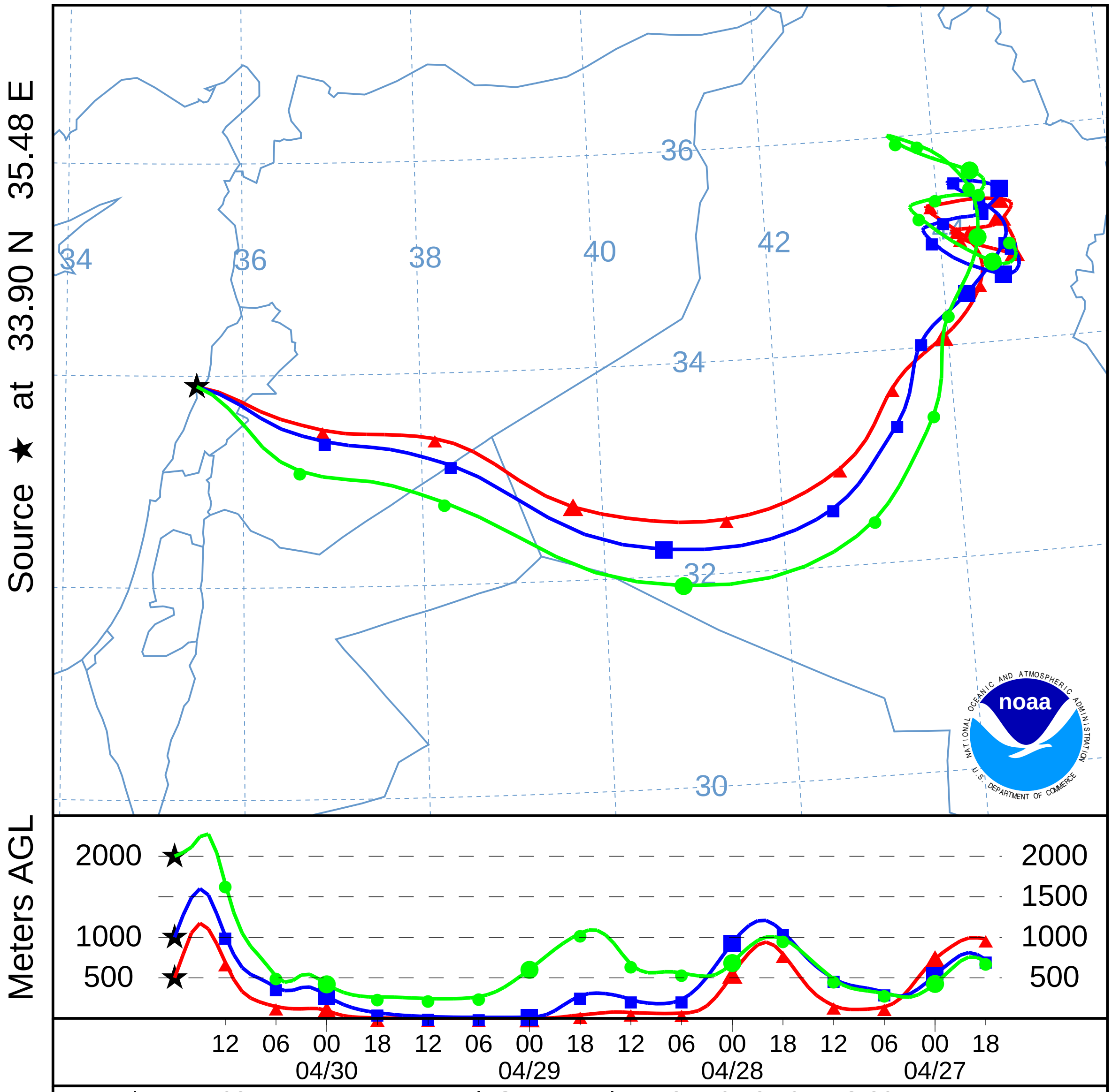

Job ID: 14084 Job Start: Wed Jun 24 10:48:49 UTC 2015
Source 1 lat.: 33.902000 lon.: 35.479000 hgts: 500, 1000, 2000 m AGL

Trajectory Direction: Backward Duration: 96 hrs
Vertical Motion Calculation Method: Model Vertical Velocity
Meteorology: 0000Z 29 Apr 2012 - GDAS1

NOAA HYSPLIT MODEL

Backward trajectories ending at 1500 UTC 02 May 12

GDAS Meteorological Data

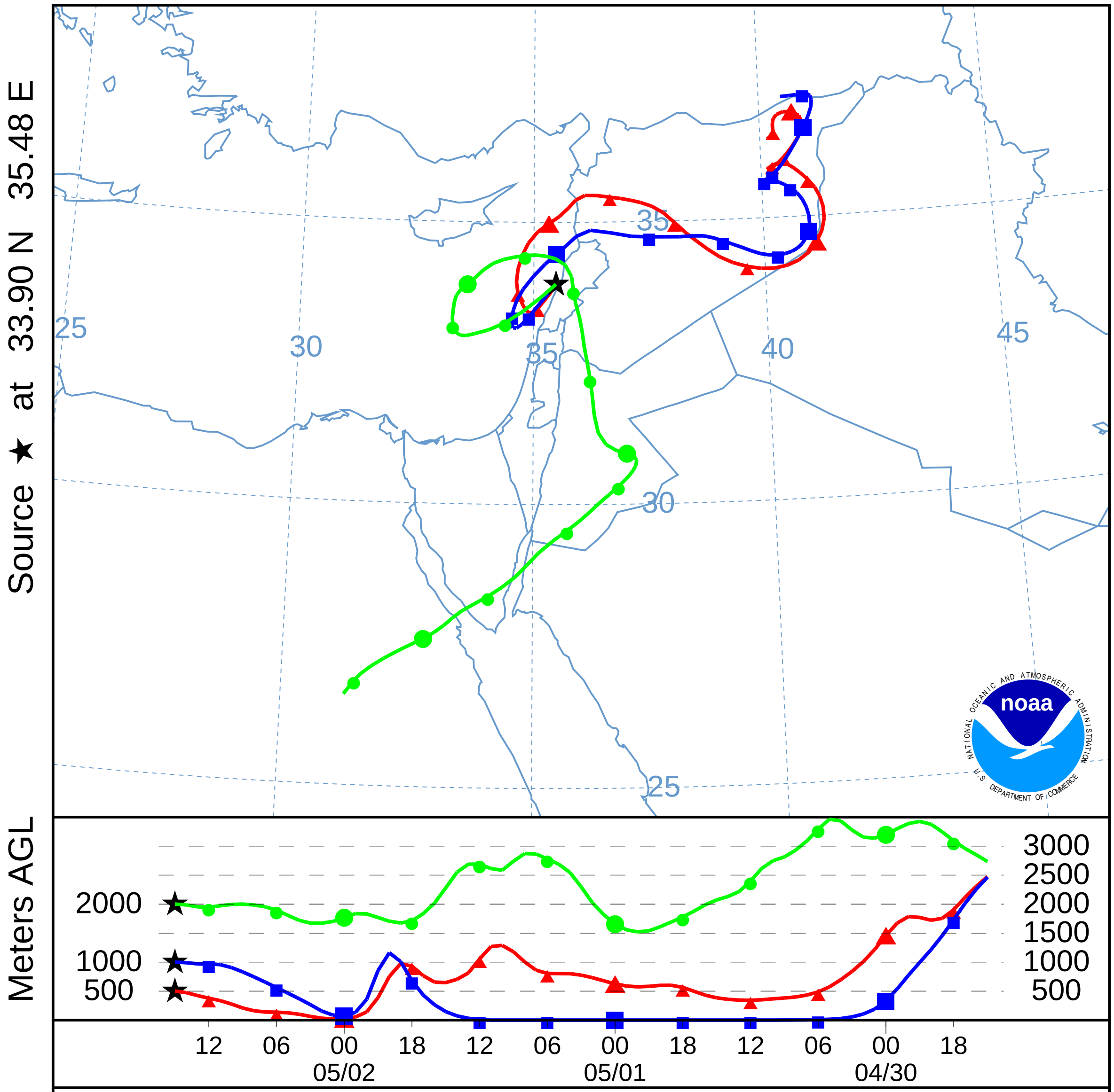

Job ID: 183969 Job Start: Tue Jun 23 13:33:07 UTC 2015
Source 1 lat.: 33.902000 lon.: 35.479000 hgts: 500, 1000, 2000 m AGL

Trajectory Direction: Backward Duration: 72 hrs
Vertical Motion Calculation Method: Model Vertical Velocity
Meteorology: 0000Z 1 May 2012 - GDAS1

NOAA HYSPLIT MODEL
Backward trajectories ending at 0600 UTC 29 May 12
GDAS Meteorological Data

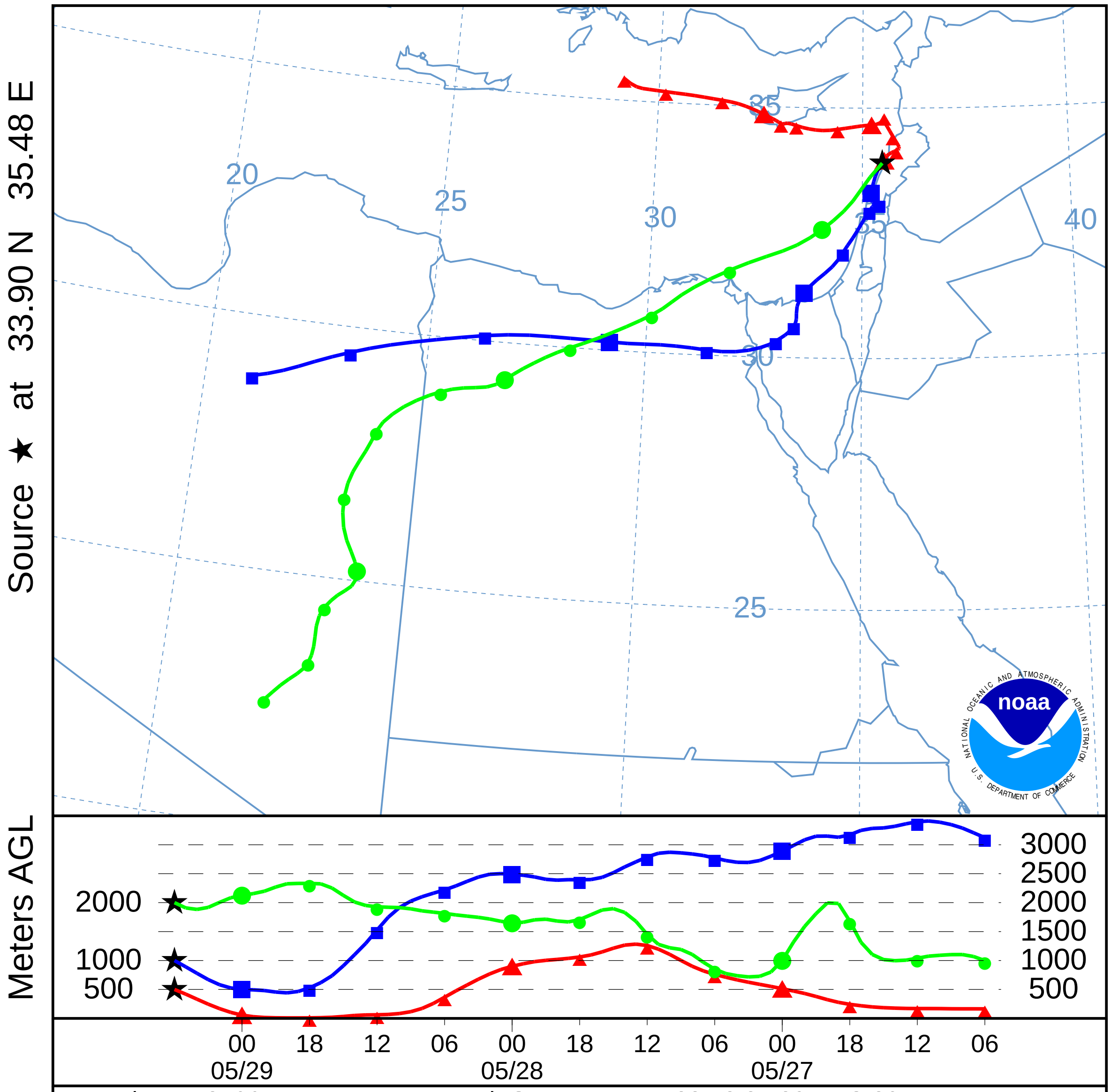

Job ID: 184064 Job Start: Tue Jun 23 13:37:29 UTC 2015
Source 1 lat.: 33.902000 lon.: 35.479000 hgts: 500, 1000, 2000 m AGL

Trajectory Direction: Backward Duration: 72 hrs
Vertical Motion Calculation Method: Model Vertical Velocity
Meteorology: 0000Z 29 May 2012 - GDAS1

NOAA HYSPLIT MODEL

Backward trajectories ending at 0000 UTC 20 Dec 12

GDAS Meteorological Data

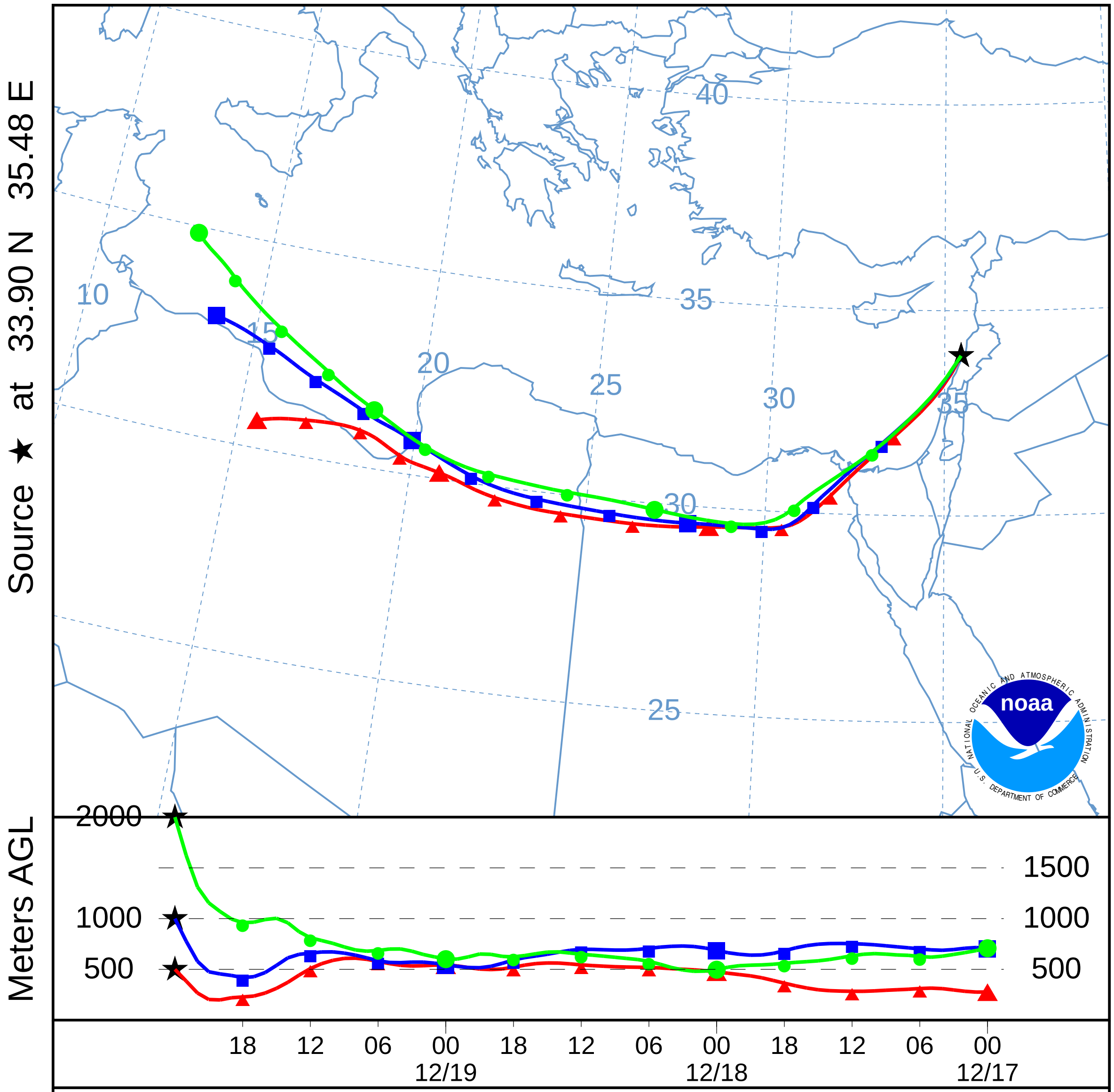

Job ID: 184122 Job Start: Tue Jun 23 13:40:28 UTC 2015
Source 1 lat.: 33.902000 lon.: 35.479000 hgts: 500, 1000, 2000 m AGL

Trajectory Direction: Backward Duration: 72 hrs
Vertical Motion Calculation Method: Model Vertical Velocity
Meteorology: 0000Z 15 Dec 2012 - GDAS1

NOAA HYSPLIT MODEL

Backward trajectories ending at 0000 UTC 21 Dec 12

GDAS Meteorological Data

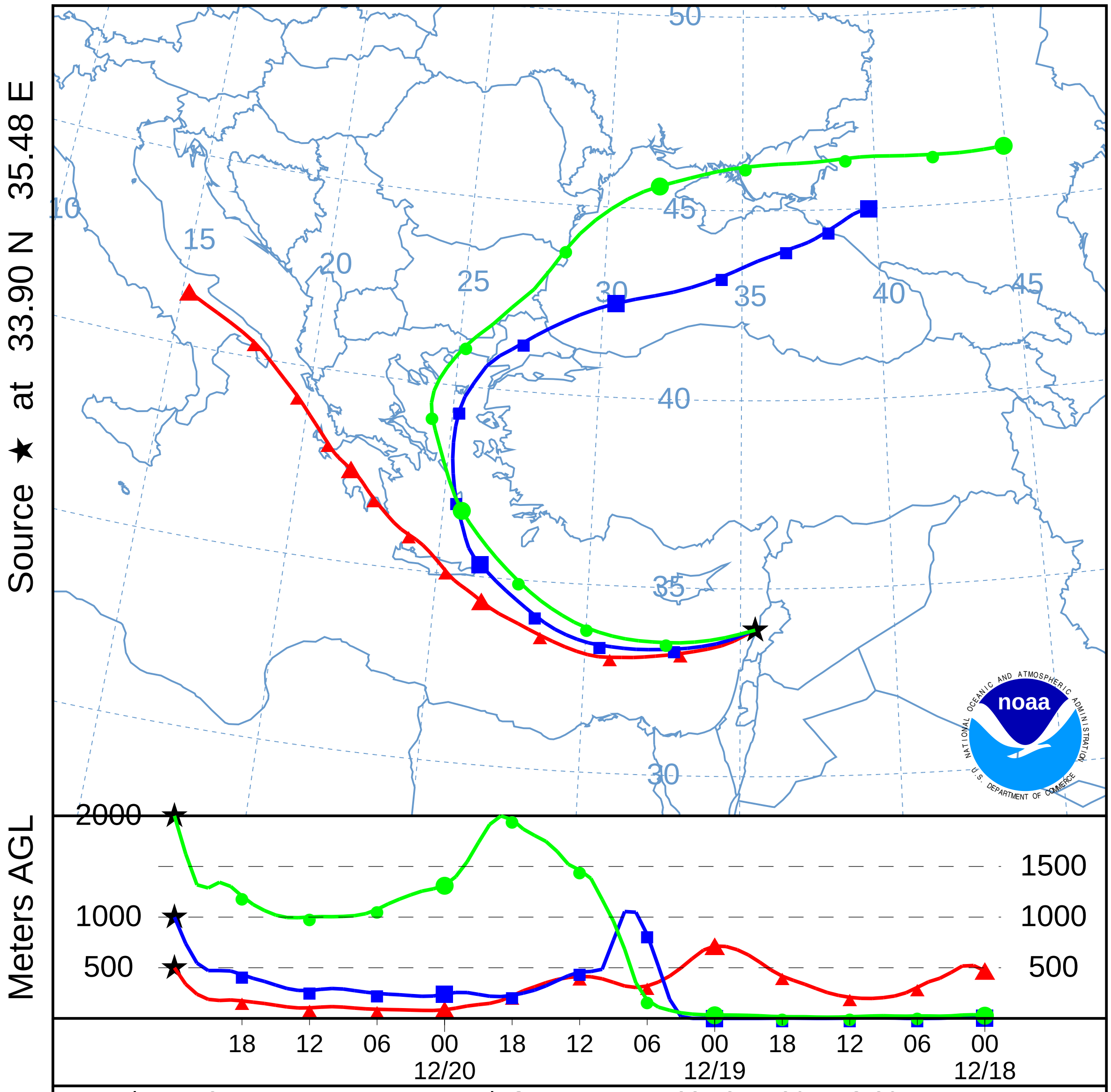

Job ID: 184145 Job Start: Tue Jun 23 13:41:36 UTC 2015
Source 1 lat.: 33.902000 lon.: 35.479000 hgts: 500, 1000, 2000 m AGL

Trajectory Direction: Backward Duration: 72 hrs
Vertical Motion Calculation Method: Model Vertical Velocity
Meteorology: 0000Z 15 Dec 2012 - GDAS1

Dust Rains Deliver Diverse Assemblages of Microorganisms to the Eastern Mediterranean Supplementary Figure S2. Sample Beirut Wind Backward Trajectories at three altitudes: 500, 1000; and 2000 m, by the nominal dates of Tables 1-3. Trajectories are for 72 hours ending at the described date and time. Red traces with triangles follow the air volume at 500 m; blue traces are for 1000 m; green traces are for 2000 m. Symbols mark 3-hour intervals with large symbols at 24-hour intervals. Each trace is labelled by the time and date at the end of the trajectory. (a) 23 September 2011. (b) 24 September 2011. (c) 1 October 2011. (d) 25 October 2011. (e) 26 October 2011. (f) 03 November 2011. (g) 14 November 2011. (h) 15 November 2011. (i) 24 December 2011. (j) 11 January 2012. (k) 13 January 2012. (l) 8 February 2012. (m) 15 February 2012. (n) 14 March 2012. (o) 2 April 2012. (p) 20 April 2012. (q) 30 April 2012. (r) 2 May 2012. (s) 29 May 2012. (t) 20 December 2012. (u) 21 December 2012. The images were obtained using the online HYSPLIT model from the US National Oceanic and Atmospheric Administration using Global Data Assimilation System meteorological data (http://ready.arl.noaa.gov/HYSPLIT_traj.php).

2011.09.23
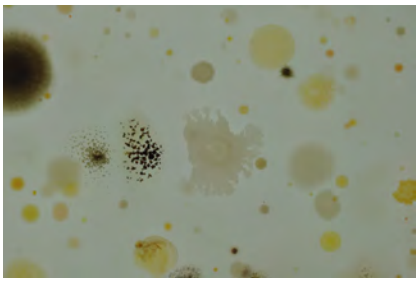
2011.09.24
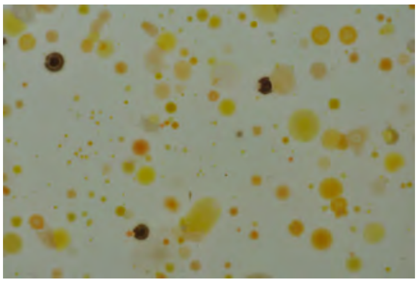
2011.10.01
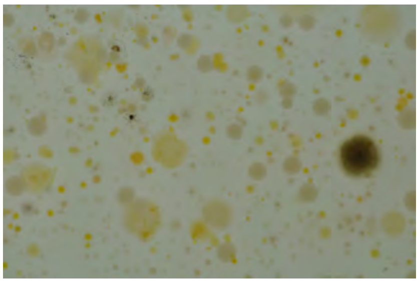
2011.10.25
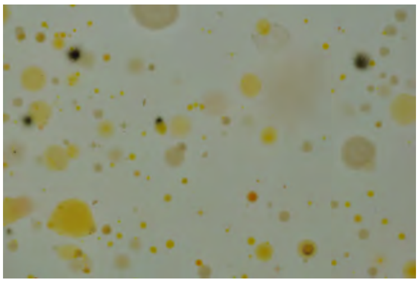
2011.10.26
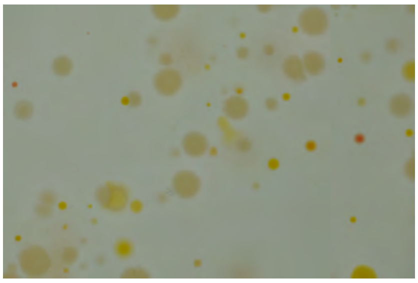
2011.11.03
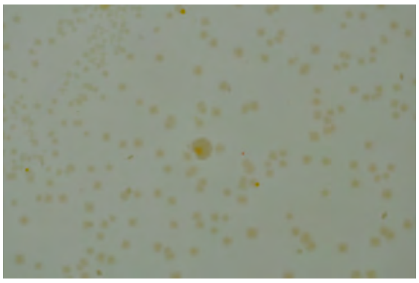
2011.11.14
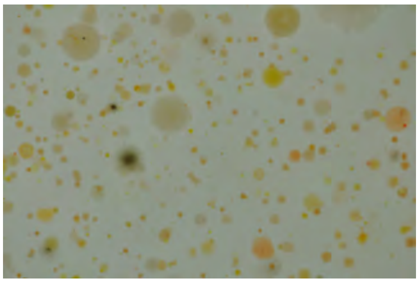
2011.11.15
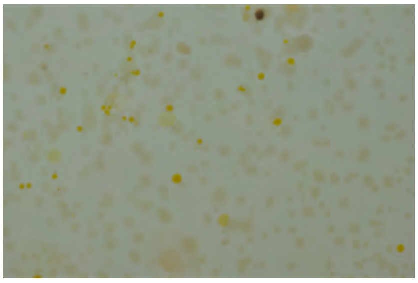
2011.12.24
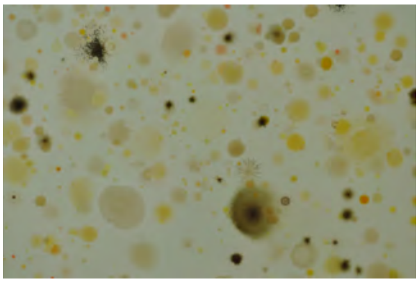
2012.01.11
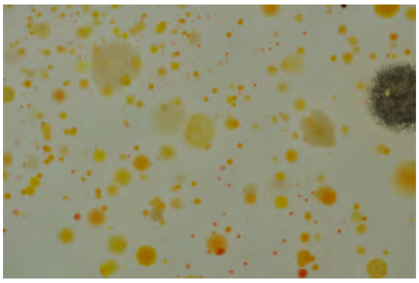
2012.01.13
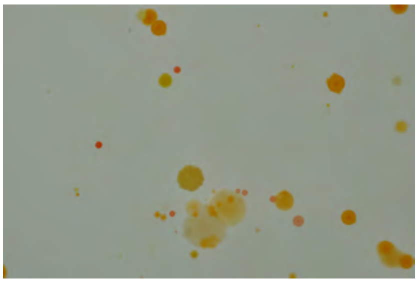
2012.02.08
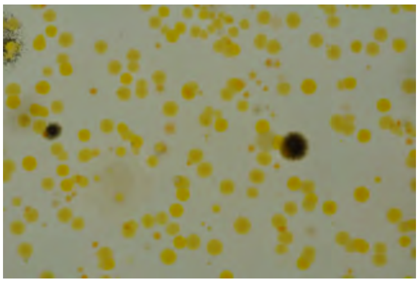
2012.02.15
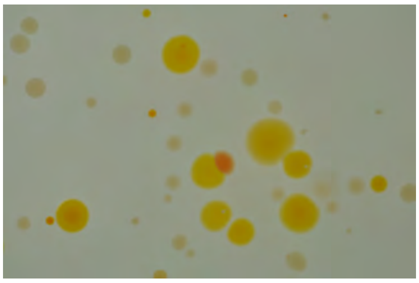
2012.03.14
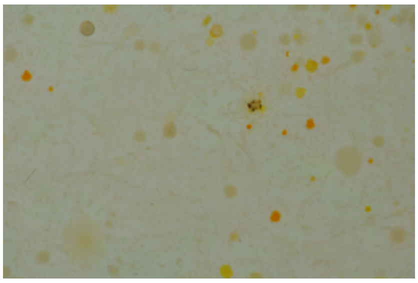
2012.04.02
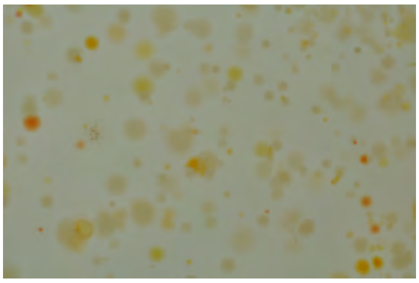
2012.04.20
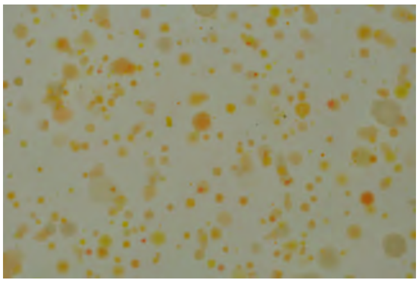
2012.04.30
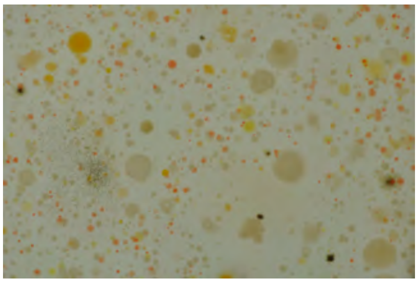
2012.05.02
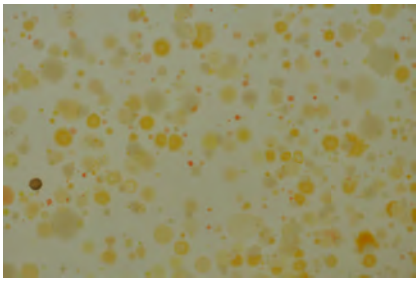
2012.05.29
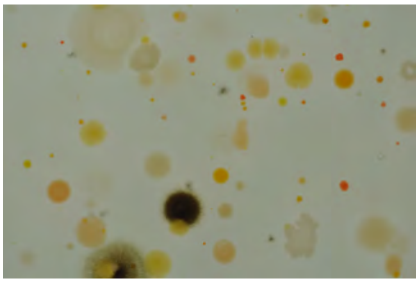
2012.12.20
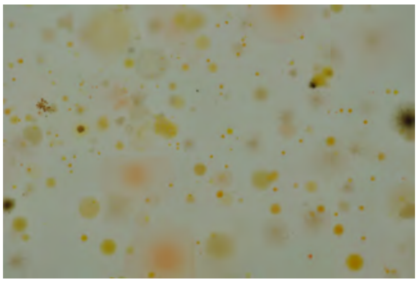
2012.12.21
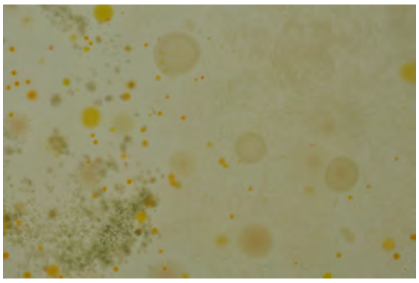

Dust Rains Deliver Diverse Assemblages of Microorganisms to the Eastern Mediterranean

Supplementary Figure S3. Cultured rain microbes. Rain residues were cultured on Reasoner's 2A agar at room temperature. Each image is labelled above with the nominal date of the rain (YYYY.MM.DD) and represents approximately 14.5 square centimetres (about 29 ×48 mm, one-quarter of the surface of a standard 85 mm diameter plate).

Dust Rains Deliver Diverse Assemblages of Microorganisms to the Eastern Mediterranean Supplementary Table S1 Number of bacterial sequences by phyla in grouped sets

sources of bacteria	sum	Actino[a]	Bacter	Cyano	Deino	Firmi	Alpha	Beta	Delta	Epsilon	gamma
all dust rains	200	31	15	4	1	27	36	73	1	3	9
all clean rains	57	9				5	2	39			2
uncult. dust rains[b]	131	7	6	4		13	32	63	1	3	2
uncult. clean rains	33							33			
all NAf-Med dust rains[c]	117	24	14	1	1	24	20	21	1	3	8
all other dust rains[d]	83	7	1	3		3	16	52			1
all clean rains	57	9				5	2	39			2
uncult. NAf-Med dust rains	60	6	6	1		13	16	12	1	3	2
uncult. other dust rains	71	1		3			16	51			
uncult. clean rains	33							33			
all 20111224 dust rains	36	5	3			8	13	2	1	3	1
all 20111115 clean rains	33	1					1	30			1
uncult. 20111224 dust rains	32	4	2			6	13	2	1	3	1
uncult. 20111115 clean rains	28							28[e]			

[a] Identification abbreviations are Actino: Actinobacteria, Alpha: Alphaproteobacteria, Bacter: Bacterioidetes, Beta: Betaproteobacteria, Cyano: Cyanobacteria , Deino: Deinococcus-Thermus, Delta: Deltaproteobacteria, Epsilon: Epsilonproteobacteria, Firmi: Firmicutes, Gamma: Gammaproteobacteria.

[b] Uncult. refers to sequences obtained only by analysis of 16S rDNA in total DNA.

[c] NAf-Med dust rains as described in Table 1, whose wind backward trajectories passed over North Africa or the Mediterranean Sea.

[d] Dust rains whose wind backward trajectories did not pass over North Africa or the Mediterranean Sea.

[e] 17 Herbaspirillum, 11 Duganella)
